# IL-1β and associated molecules as prognostic biomarkers linked with immune cell infiltration in colorectal cancer: an integrated statistical and machine learning approach

**DOI:** 10.1007/s12672-025-01989-3

**Published:** 2025-02-28

**Authors:** Karishma Sahoo, Vino Sundararajan

**Affiliations:** Integrative Multiomics Lab, School of Bio Sciences and Technology, Vellore Institute of Technology, Vellore, Tamil Nadu 632014 India

**Keywords:** Prognosis, Diagnosis, Immune cell infiltrate, Aberrant methylation, Microarray, Biomarker identification

## Abstract

**Purpose:**

Colorectal cancer (CRC) is the third most common cancer globally, necessitating novel biomarkers for early diagnosis and treatment. This study proposes an efficient pipeline leveraging an integrated bioinformatics and machine learning framework to enhance the identification of diagnostic and prognostic biomarkers for CRC.

**Methods:**

A selection of methylated differentially expressed genes (MeDEGs) and features (genes) was made using both statistical and Machine learning (ML) approaches from publically available datasets. These genes were subjected to STRING network construction and hub genes estimation, separately. Also, essential miRNAs (micro-RNAs) and TFs (Transcription factors) as regulatory elements were revealed and findings were validated through scRNA-seq analysis, promoter methylation, gene expression levels correlated with pathological stage, and interaction with tumor-infiltrating immune cells.

**Results:**

Through an integrated analysis pipeline, we identified 27 hub genes, among which CTNNB1, GSK3B, IL-1β, MYC, PXDN, TP53, EGFR, SRC, COL1A1, and TGBF1 showed better diagnostic behaviour. Machine learning approach includes the development of K-Nearest Neighbors (KNN), Artificial Neural Networks (ANN), and Random Forest (RF) models using TCGA datasets, achieving an accuracy range between 99 and 100%. The Area Under the Curve (AUC) value for each model is 1.00, signifying good classification performance. The high expression of some diagnostic genes was associated with poor prognosis, concluding *IL-1β* as both a prognostic and diagnostic biomarker. Additionally, the NF-κB and microRNAs (miR-548d-3p, miR-548-ac) and TFs (NFκB and STAT5A) play a major role in the comprehensive regulatory network for CRC. Furthermore, hub genes such as IL-1β, TGFB1, and COL1A1 were significantly correlated with immune infiltrates, suggesting their potential role in CRC progression.

**Conclusion:**

Overall, the elevated expression of IL-1β coupled with abnormal DNA methylation, and its consequent effect on the PI3K/Akt signaling pathway are relevant prognostic and therapeutic marker in CRC. Additional molecular candidates reveal insights into the epigenetic regulatory targets of CRC and their association with immune cell infiltration.

**Supplementary Information:**

The online version contains supplementary material available at 10.1007/s12672-025-01989-3.

## Highlights

- Utilizing an integrative bioinformatics approach, our research delves into DNA methylation, transcriptomics, and microarray datasets analysis to identify potential epigenetic and genomic biomarkers crucial for early diagnosis, prognosis, and treatment of CRC.
- Our findings highlight IL-1β as an effective diagnostic and prognostic biomarker for CRC, predicting immune cell infiltration. Additionally, the expression of IL-1β, IL-6, and TNF-α also correlates with inflammatory cell infiltration, indicating their potential role in CRC progression.
- We propose a hypothesis suggesting a cyclical regulatory mechanism involving GSK3β, AKT1, PI3K, EGFR/ERBB2, PTEN, TP53, and TGFβ1 as derived Hub genes, with NFκB and STAT5A as transcription factors, and the interplay of microRNAs such as miR 548d-3p, miR 548 ac, and miR 181a. These effective diagnostic hub genes and their regulatory molecules engage in mutual activation and regulation, ultimately driving the activation of the PI3K/AKT signaling pathway.

**Supplementary Information:**

The online version contains supplementary material available at 10.1007/s12672-025-01989-3.

## Introduction

Colorectal cancer (CRC) stands as a formidable global health challenge and the second leading cause of cancer-related mortality worldwide. The year 2023 alone witnesses a staggering estimate of 153,020 new CRC cases, with 52,550 individuals dying of this disease [1]. The incidence is expected to rise by 60% by 2030, with over 2.2 million new cases and 1.1 million deaths [2]. Early detection methods for CRC include fecal-based, enteroscopy, surgical and multimodal therapy, and blood-based examinations, despite which the disease shows poor prognosis and late detection [3]. The last two decades have seen a shift from traditional chemotherapy to targeted therapies which have unveiled a series of biomarker genes for developing precise diagnostic and prognostic markers [4]. Therefore, it is crucial to accurately identify the specific molecular mechanism involved in the onset, growth, and metastasis of CRC.

**Fig. 1:**
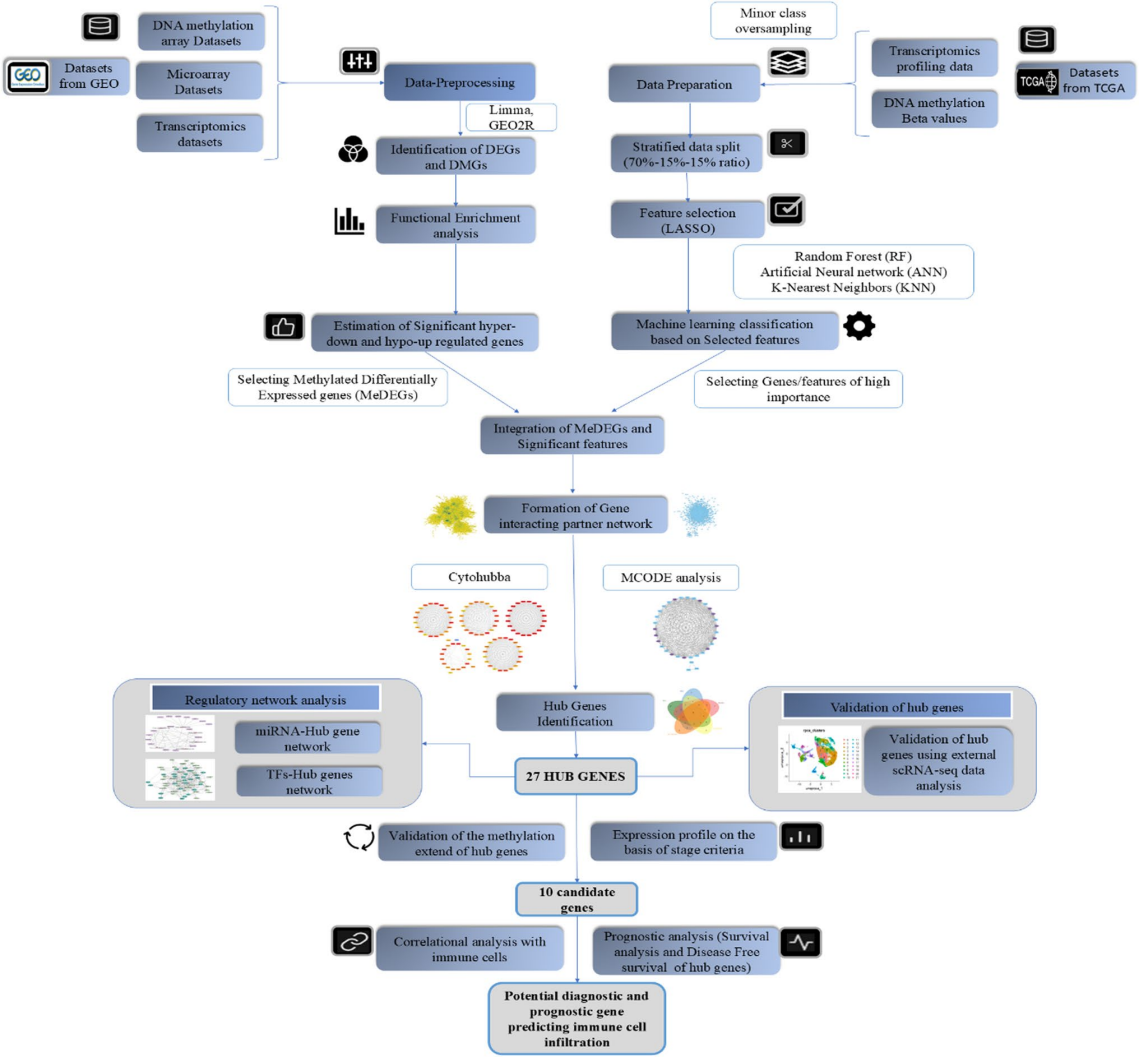
Methodological workflow of the study: The analysis integrates statistical and ML approaches across diverse biological data types (DNA methylation, microarray, and RNA-seq). The statistical approach retrieves differential genes from the GEO database, while the ML approach focuses on the TCGA database. Integrated MeDEGs and significant features identify 27 hub genes, establishing them as potential CRC biomarkers. These genes undergo validation of promoter methylation; stage-based expression profiling and Regulatory network analysis deriving candidate genes showing an elevated expression. Candidate genes are analyzed for prognostic and correlational relationships with immune cells to identify molecular signatures with diagnostic and prognostic potential, establishing them as therapeutic targets related to immune infiltration in CRC

Precision medicine is revolutionizing healthcare through emerging technologies like genome and transcriptome sequencing, artificial intelligence, and gene-editing technology [5], and in the context of pharmaceutical treatments, distinct gene sets may influence individual responses, making bioinformatics and some supervised machine-learning algorithms crucial for discovering disease-associated biomarkers [6, 7]. The collective analysis of different biological data can yield significant insights into molecular mechanisms and enhance our understanding of biological systems and their complexities [8]. Moreover, biomarkers influence immune infiltration levels and trigger immune responses by regulating the tumor microenvironment (TME), which is crucial for cancer progression and affects the outcome of immunotherapy. Hence, this study aims to identify reliable molecular mechanisms in CRC progression, focusing on diagnostic and prognostic characteristics related to immune infiltration, which significantly influence tumor behaviour, treatment response, and patient outcomes. The integration of gene expression and DNA methylation profiling data analysis, bioinformatics techniques, and ML algorithms will enable the screening of appropriate biomarkers and guide therapeutic, diagnostic, and treatment choices [9]. To achieve this, we employed an integrative statistical and machine learning approach to analyze datasets (including different biological datatypes) separately from GEO and TCGA, respectively. This dual approach leverages extensive datasets from the most reliable public repositories to provide a comprehensive understanding of CRC etiopathogenesis, while also establishing a robust framework for identifying disease-specific molecular biomarkers. Our findings also reveal a regulatory network of miRNAs and transcription factors (TFs) with hub genes, exploring the candidate pathophysiological mechanisms underlying CRC development and thus providing novel therapeutic and diagnostic avenues. Additionally, the prognostic, survival and correlational analysis with the immune cells also provided the theoretical basis for the development of improved immunotherapy treatment strategies for CRC. Figure 1 depicts the comprehensive computational pipeline utilized in our study, integrating both statistical and machine-learning approaches.

## Material and methods

In this study, we employ a dual approach—statistical and machine learning—to derive differential genes from publicly available datasets. The statistical approach focuses on datasets retrieved from the Gene Expression Omnibus (GEO), while the machine learning approach utilizes datasets from The Cancer Genome Atlas (TCGA) portal. To enhance the robustness of our findings, we integrate the differential genes identified from both GEO and TCGA analyses, providing a comprehensive set of genes for further processing in the identification of biomarkers through subsequent methods (Fig. 1).

### Dataset construction and preprocessing

We employed a vertical data integration analysis approach to directly and concurrently integrate individually generated results from differential analysis done on various data types. Microarray, transcriptomics, and DNA methylation array data were retrieved from the NCBI-GEO platform using the “GEOquery” R package*.* Additionally, the CRC mRNA expression and DNA methylation array datasets (27 K and 450 K) were downloaded from NIH-GDC portal, using the “TCGAbiolinks” R package. Table 1 outlines the filters/keywords used to retrieve different types of datasets from public repositories, while *Supplementary Table 1* provides the GEO accession IDs of datasets and their corresponding metadata, included in this study. Studies involving treated samples were excluded, while those performed on *Homo sapiens* with a minimum of six samples were included. The sample distribution from the TCGA database can be visualized in *Supplementary Fig. 4A* and *Supplementary Fig. 4B.* The construction of TCGA datasets involved the integration of both COAD and READ datasets, to form the respective combined transcriptomics and methylation datasets separately. Briefly, the gene expression and DNA methylation datasets from GEO, were retrieved for each of the studies using the “fData” function, and rows with missing values were omitted. Moreover, in methylation datasets, the probes attached to the sex chromosomes (chr X and chr Y) were eliminated using the package “IlluminaHumanMethylation450kanno.ilmn12. hg19” in R. The preprocessing of datasets obtained from the TCGA portal involved similar methods, supplemented by the inclusion of sample labels categorized as diseased or normal, mapped to the corresponding expression data for both transcriptomic and methylation array analysis. Additionally, gene and CpG probe IDs were annotated with official gene symbols using the AnnotationDbi, org.Hs.eg.db, and IlluminaHumanMethylation450kanno.ilmn12.hg19 Bioconductor packages.

**Table 1:** The filters/keyword used for retrieving different type of datasets from the public repositories i.e., TCGA and GEO database

Database	Inclusion/exclusion Criteria	Keywords and filters	Microarray	RNA-seq	DNA methylation
GEO	The inclusion criteria: (I) Inclusion of samples from both diseased human cancer tissue and adjacent normal tissue; (II) Selection of the datasets from original experiment conducted. Exclusion criteria: (I) Avoiding data from cell lines or animal experiments and treatment against the targeted pathways; (II) Studies focussing on rectal cancer or caecum cancer	Keyword: colorectal cancer; colon AND rectal cancer; colorectal AND normal; Filter: selected species as “Homo sapiens” and study types as “Methylation profiling by array”, “Expression profiling by array” and “Expression profiling by High throughput sequencing”	12 datasets	13 datasets	21 datasets
Database	Inclusion/exclusion Criteria	Keywords and Filters	Transcriptomics		DNA methylation
TCGA	Data category: DNA methylation and transcriptome profiling. Data availability: Open Access	Keywords: Colorectal cancer Filters for Data type: Gene Expression Quantification and methylation beta values; Platform: Illumina Human Methylation 27 K and Illumina Human Methylation 450 K	699 CRC samples (51 normal and 648 tumour)		861 CRC samples (119 normal and 742 tumor)

### Statistical approach

This approach includes analyzing data obtained from the GEO database.

#### Differential expression analysis

Differentially Expressed Genes (DEGs) and differentially methylated CpG sites (DMS) were obtained from microarray and DNA-methylation array datasets, respectively, using the “limma” package [10]. Additionally, DEGs were identified from transcriptomics datasets using the ‘GEO2R’ tool”. These differential genes exhibit distinct expression levels between tumor and normal samples. Conceptually, hypermethylated CpG sites are associated with upregulated genes, while hypomethylated CpG sites are linked to downregulated genes. As part of the normalization procedure, any outliers are eliminated through the application of log2 transformation. Additionally, the default approach for correcting the p-value is the Benjamin Hackenberg method. The threshold used was p.value < 0.05 and logFC > 1 for upregulated DEGs; logFC < 1 for downregulated DEGs, and |logFC|> 0.5. Similarly, DMS was determined with FDR < 0.05 and was considered as the threshold and the gene matching file with the CpG locus came from the Illumina website. Subsequently, the “ggplot” package was used for creating volcano plots as the visual representation of the upregulated, downregulated, and non-significant genes derived from each dataset. Venn diagram was generated using the “matplotlib” python package to visualize the intersection between DEGs and DMS. The common genes derived from downregulated DEGs in both gene expression and transcriptomics analysis along with hypermethylated DMGs belong to MODULE I genes whereas common genes that are upregulated in both gene expression and transcriptomics analysis along with hypomethylated DMGs belong to MODULE II genes. The gene duplicates were removed.

#### Functional enrichment of gene sets belonging to modules

The derived gene sets from MODULE I and II were interpreted using DAVID’s functional annotation tool including pathway analysis and gene ontology analysis [11]. Biological pathways with adjusted p-values and FDR below 0.05 were considered statistically significant. Common genes were manually curated to select only cancer-related gene ontology terms and pathways for further analysis. Later, genes satisfying both these criteria were subsequently designated as methylated differentially expressed genes (MeDEGs). The visualization of results was done using the dot plot for both GO and KEGG pathway analysis using “ggplot2”, “tidyverse”, and “dplyr” packages.

### Machine learning approach

This approach includes analyzing data obtained from the TCGA- GDC portal.

#### Class imbalance

TCGA-GDC database shows the non-uniform distribution of the samples (normal and tumor) which results in biased ML model development with incorrect identification of instances of the minor class leading to misleading accuracy [12]. To address this, re-sampling techniques (both undersampling and oversampling) are employed to fine-tune the balance and ensure a more equitable class distribution. In this case, an oversampling technique such as SMOTE (Synthetic Minority Oversampling Technique) is typically preferred instead of undersampling, which is a popular data augmentation technique that generates synthetic minority class instances by randomly selecting a minority class instance and its k nearest neighbors. This results in doubling the number of minority class samples after application [13].

#### Dataset splitting using K-fold cross-validation

Initially, the datasets were divided into three segments: training, validation, and test sets, in a 70:15:15 ratio. Before training ML models, the training set was subjected to K-fold cross-validation, while the test set remained held out. Later, the unseen data was used to predict the accuracy rate of the trained models. K-fold cross-validation is a robust approach for evaluating the effectiveness and generalizability of ML models by partitioning the dataset into k equally sized subsets or folds. Iteratively, the model is trained on k-1 folds while the remaining fold serves as the validation set. This process repeats k times, ensuring each fold acts as the validation set exactly once. The overall model performance is determined by averaging the validation scores across all iterations. This technique is done k times with a different fold as the test set each time, which can be mathematically denoted as $k= \frac{N}{\alpha }$ , and also$t=N-T$. Here, *N* represents the total data points; α denotes the fold size, and *k* indicates the number of folds. The training size(*t*) is derived by subtracting the test size (*T*) from *N* [14]. Even more, data leakage occurs when information from the hold-out test set leaks into the training dataset. Certainly, data leakage issues can be identified by observing high accuracy on the training and validation sets, but significantly lower accuracy when tested on the test set. To check for data leakage issues, cross-validation can be applied by keeping the test set separate and unaltered, which is later used to evaluate the performance of the three models (RF, ANN, KNN) separately. The implementation of K-Fold Cross-Validation was done using the Scikit-Learn framework.

#### Feature selection

The CRC gene expression dataset contained 68,575 features (genes) for 699 samples whereas methylation profiling datasets (with a combined 450 k and 27 k) contained 16,384 features each for 861 samples. The LASSO (The Least Absolute Shrinkage and Selection Operator) algorithm is a regularization technique that enhances the functionality of linear regression models and effectively selects a subset of features from a dataset with numerous features [15]. It penalizes the regression coefficients with L1 distance and most coefficients are reduced to zero. We employed the scikit-learn module [16] to implement the LASSO algorithm and identify significant features by selecting non-zero inputs.

#### Machine learning (ML) based classification model

ML approaches hold immense potential to revolutionize cancer research and treatment by uncovering novel insights into the mechanisms of oncogenesis. This results in the development of more accurate and effective diagnostic, prognostic, and predictive markers for various cancers, including CRC. The Random Forest (RF) algorithm demonstrates superior performance in classifying cancer tumor data by constructing an ensemble of decision trees during the training phase. This supervised ML algorithm uses multiple-level randomization procedures for the classification task, resulting in the construction of an individual decision tree for each sample. For each tree node, an RF algorithm selects a random subset of variables and uses only those to determine the optimal split. Later, it determines the final class which is based on the majority voting from the ensemble of decision trees [17]. The model delivers high accuracy and efficiency for large datasets, even when a significant amount of data is missing. A critical assumption for the effective functioning of the algorithm is as follows: (i) To ensure accurate predictions, the dataset's feature variables must possess actual values instead of imputed estimates. (ii) The predictions derived from each tree should display minimal correlations [18]. K-nearest neighbors (KNN) is a non-parametric method that identifies the k nearest neighbors of a new sample based on distance metrics, followed by assigning to the most frequent class among its neighbors [19]. The algorithm operates by calculating the Euclidean distance between points, sorting these distances, and selecting the k-nearest points to determine the target class based on majority voting. Euclidean distance is commonly used to calculate distances between samples in KNN algorithm which can be mathematically represented as follows:

$$distance\left({s}_{1},{s}_{2}\right)= \sqrt{\sum_{j=1}^{p}{\left(g1j-g2j\right)}^{2}}$$

where s_1_ and s_2_ are the two different samples whereas p is the total number of genes in expression data. Selecting an optimal k is critical, as larger values can mitigate noise but may lead to underfitting. However, irrelevant features or noise can degrade performance, highlighting the need for effective feature selection and data preprocessing to maintain accuracy [20]. Artificial neural networks (ANNs) are multi-layered models that process data through weighted connections and its architecture includes the number of input and output layers as well as one or more hidden layers, depending on the dataset’s covariates and outcome classes [21]. The nodes of the input layer receive data as a vector of predictor variables which is then passed to the first hidden layer. Subsequently, the input is adjusted using weights, summed, and passed through a non-linear activation function (e.g., sigmoid or hyperbolic tangent) in the hidden layer to generate outputs. The output for each neuron is computed as ${v}_{k}= \sum_{i=1}^{n}{w}_{ki}{x}_{i}$ and ${y}_{k}= \varphi \left({v}_{k}+{v}_{{k}_{0}}\right)$ where *x*_1_, *x*_2_…*x*_n_ are the input signals in the neuron (*k*) and the weights connecting to the neuron are denoted as* ω*_*k*1_, *ω*_*k*2_…*ω*_*k*n_ [22]. In this context, ${v}_{k}$ represents the net input signal; ${y}_{k}$ denotes the neuron’s output, *v*_*k*0_ is a bias term and ϕ(.) is the sigmoid activation function, defined as follows:

$$\varphi \left(v\right)= \frac{1}{1+ {e}^{-v}}$$

This study uses machine learning algorithms like Random Forest, KNN, and ANN to create classification models for cancer research, by utilizing Python-based sklearn and TensorFlow framework. The performance evaluation involved calculating accuracy, training time, and classification scores for the best-performing algorithm, with learning curves accessing the performance of the ANN model. Further, the classification metric evaluates the performance of a classification-based ML model by showcasing its precision, recall, F1 score, and support. Classification metrics include True Positives (*TP*), True Negatives (*TN*), False Positives (*FP*), and False Negatives (*FN*). The proportion of *TP* compared to the sum of true and false positive results, is defined as precisionT. Moreover, recall can be stated as the ratio of true positive results with that of the summation of true positive and false negative results. Accuracy score is defined to be the proportion of correct predictions relative to the total number of predictions made and can be stated as Accuracy = $\frac{TP+TN}{TP+TN+FP+FN}$.

### Network analysis and visualization of the functional interactions and protein–protein interaction network (PPI network) with selection of hub genes

The functional interactions between all the differential genes, amalgamated from both statistical and ML approaches, were evaluated using the protein interaction database (STRING) [23]. Cytoscape (version 3.10.1) facilitated the visualization of the PPI network derived from STRING results, while MCODE and Cytohubba identified densely connected regions within the constructed network [24]. The parameters used in the analysis were as follows: MCODE scores > 5, degree cut-off = 2, node score cut-off = 0.2, max depth = 100, and k-score = 2[25]. Also, Cytohubba was used to find hub genes in the constructed network, using 5 different methods consisting of 3 local-based methods (MCC, MNC, and Degree) and 2 global-based methods (betweenness and closeness i.e., based on the shortest path). The top twenty genes from each of these methods were extracted and the genes showing their presence in three or more than three scoring methods are considered to be hub genes. Next, genes appearing in at least two scoring methods of Cytohubba and are also found in the MCODE clusters with the highest three cluster scores were identified as biomarker hub genes.

### Regulatory network analysis of Hub genes

Both computational and experimental analyses indicate that miRNA and TFs have a regulatory effect on protein-coding genes. Several tools and databases can be used for identifying miRNAs and TFs showing their ability to bind to target genes, functioning as an oncogene or tumour suppressors. To identify key micro-RNAs (miRNAs) as the post-transcriptional regulators of Hub genes, the Hubgene-miRNAs interaction network was constructed after comprehending the miRNAs of each hub gene from the miRDB database [26]. The interaction between the hub genes and transcription factors (TFs) was investigated using Cytoscape’s iRegulon plugin. Within iRegulon, the ranking of enriched motifs was based on their direct targets, employing a position weight matrix for precision [27]. Key TFs and miRNAs regulating the targeted hub genes were identified by selecting the top-interacting miRNAs and TFs from the network.

### Validation using scRNA-seq datasets

The scRNA-seq data (GSE221575), obtained from tissues of a CRC patient, were retrieved from the Gene Expression Omnibus (GEO) database [28]. The data were generated using the Illumina NovaSeq 6000 platform. Processing of the data was conducted using the Seurat package version 3.1.1 in R version 3.6.1. Quality control involved extracting genes with at least 200 features with non-zero counts and a minimum of 3 cells. The filtered data underwent normalization through log transformation for subsequent analysis. Dimensionality reduction, t-SNE, and K-means clustering were employed during data processing, followed by the utilization of the UMAP function to visualize the placement of similar cells in low-dimensional space. Moreover, the VlnPlot function and Seurat's Featureplot function were used to determine the expression of acquainted marker genes (Hub genes) to assign clusters. Also, we used the function of FindAllmarkers that defines clusters via differential expression (DE). The scRNA-seq data were analyzed for DEGs using the R package Seurat.

### The validation of promoter methylation and other expression analysis of derived Hub genes

In this study, UALCAN [29] was utilized to extensively examine promoter DNA methylation patterns across control and CRC samples for the 27 hub genes. The beta value indicated the level of DNA methylation ranging from 0 (unmethylated) to 1 (fully methylated), validating the extent of hypomethylation or hypermethylation. Moreover, GEPIA (The Gene Expression Profiling Interactive Analysis) was utilized to evaluate the expression of the selected hub genes showing good promoter methylation levels in COAD and READ patients via the “DIY Expression” page [30]. The genes with promoter methylation validation and high expression rate are further subjected to expression stratification based on stage criteria (UALCAN). Data are mean ± SE. *P < 0.05; **P < 0.01; ***P < 0.001. The hub genes with higher expression in normal/tumour nd stage-based differential analysis along with elevated promoter methylation level are being analyzed for survival and correlational analysis.

### Prognostic analysis and correlational analysis with immune cells

The identified candidate genes were subjected to survival analysis using GEPIA, which allows users to select specific cancer types for overall or disease-free survival analysis [30]. A p-value threshold of 0.05 was applied, with Student’s t-test utilized to derive p-values for expression analysis. Hence, the median hub gene expression was used as a cutoff value to classify groups into high and low-expression groups, and the prognostic analysis was performed using a Kaplan–Meier curve. The hub genes with prognostic significance were analyzed for their correlation with immune infiltration across various malignancies using the TIMER platform [31]. This allowed us to examine their expression and correlation with tumor-infiltrating lymphocytes (TILs), including B cells, CD4 + T cells, CD8 + T cells, neutrophils, macrophages, and dendritic cells.

## Results

### Results for gene expression profiling

The analysis identified a total number of 25708 upregulated and 56100 downregulated genes (total = 224754) in transcriptomics datasets whereas derived 39408 upregulated and 89737 downregulated genes (total = 281994) from microarray datasets, without any duplicates. The detailed description of the same is discussed in Table 2. The upregulated and downregulated DEGs were displayed on the right and left sides of the volcano plot with red and blue color, respectively (showcased in *Supplementary Fig. 1* and *Supplementary Fig. 2*).

**Table 2:** The list of differentially expressed genes showing the proportion of upregulated and downregulated genes of each respective microarray and transcriptomics dataset

S.No	Accession ID	Differentially expressed genes (DEGs)	Upregulated genes	Downregulated genes
Microarray datasets analysis				
1	GSE4107	22185	3338	4691
2	GSE9348	22185	2276	10230
3	GSE10950	11413	1386	5857
4	GSE15960	22185	1658	2801
5	GSE23878	22186	669	10528
6	GSE25070	17542	1238	7097
7	GSE74602	17182	10646	3570
8	GSE89076	32079	2915	19674
9	GSE110224	22165	1763	7988
10	GSE110225	22185	3987	5444
11	GSE136735	28766	718	3423
12	GSE141174	17182	3702	2862
13	GSE156355	24739	5112	5572
Transcriptomics datasets analysis				
1	GSE200427	19664	1599	2037
2	GSE196006	18156	1671	7902
3	GSE164541	17681	1270	4234
4	GSE144259	23685	3602	12182
5	GSE142279	19548	2187	8649
6	GSE137327	17974	1397	1517
7	GSE136630	17092	1833	4771
8	GSE100243	16849	2787	2949
9	GSE95132	21436	4747	5547
10	GSE89393	15632	2466	3410
11	GSE72820	17229	160	209
12	GSE50760	19808	1989	2693

### Results for methylation analysis

The total number of genes derived from all datasets may contain duplicates, as multiple methylated sites (DMSs) can be annotated with the same gene. Therefore, the duplicated genes were removed and the data was further analyzed. The total number of hypermethylated DMGs is 108,316 and hypomethylated DMGs is 28,158 (total = 136,474). The detailed description of the same is discussed in Table 3. The volcano plots show methylation expression with red, blue, and black colors as Hypermethylated sites, Hypomethylated sites, and non-significant sites, respectively *(Supplementary Fig. 3).*

**Table 3:** The differentially methylated sites with the respective proportion of Hypermethylated and hypomethylated genes annotated with respective expressed sites

S.No	Accession ID	Differentially expressed methylated sites	Hypermethylated genes (DMGs)	Hypomethylated genes (DMGs)
1	GSE33545	25819	510	344
2	GSE25062	22519	4525	3781
3	GSE29490	26917	4378	3219
4	GSE33181	26588	2427	2002
5	GSE101764	451540	15646	29337
6	GSE119526	865859	10989	24582
7	GSE139404	464012	12795	26818
8	GSE184494	462736	12352	25441
9	GSE27130	25,048	1590	6702
10	GSE17648	27185	1510	5717
11	GSE21181	8465	1510	5717
12	GSE40055	127180	1539	4063
13	GSE47071	27473	4697	2740
14	GSE68060	148973	3701	14584
15	GSE77954	485577	11972	17108
16	GSE79740	475868	19843	11753
17	GSE98531	21642	542	9680
18	GSE129364	446496	1186	8273
19	GSE149282	827476	8742	22162
20	GSE39446	1505	399	657
21	GSE64112	1505	252	218

### Results for finding common genes

**Fig. 2:**
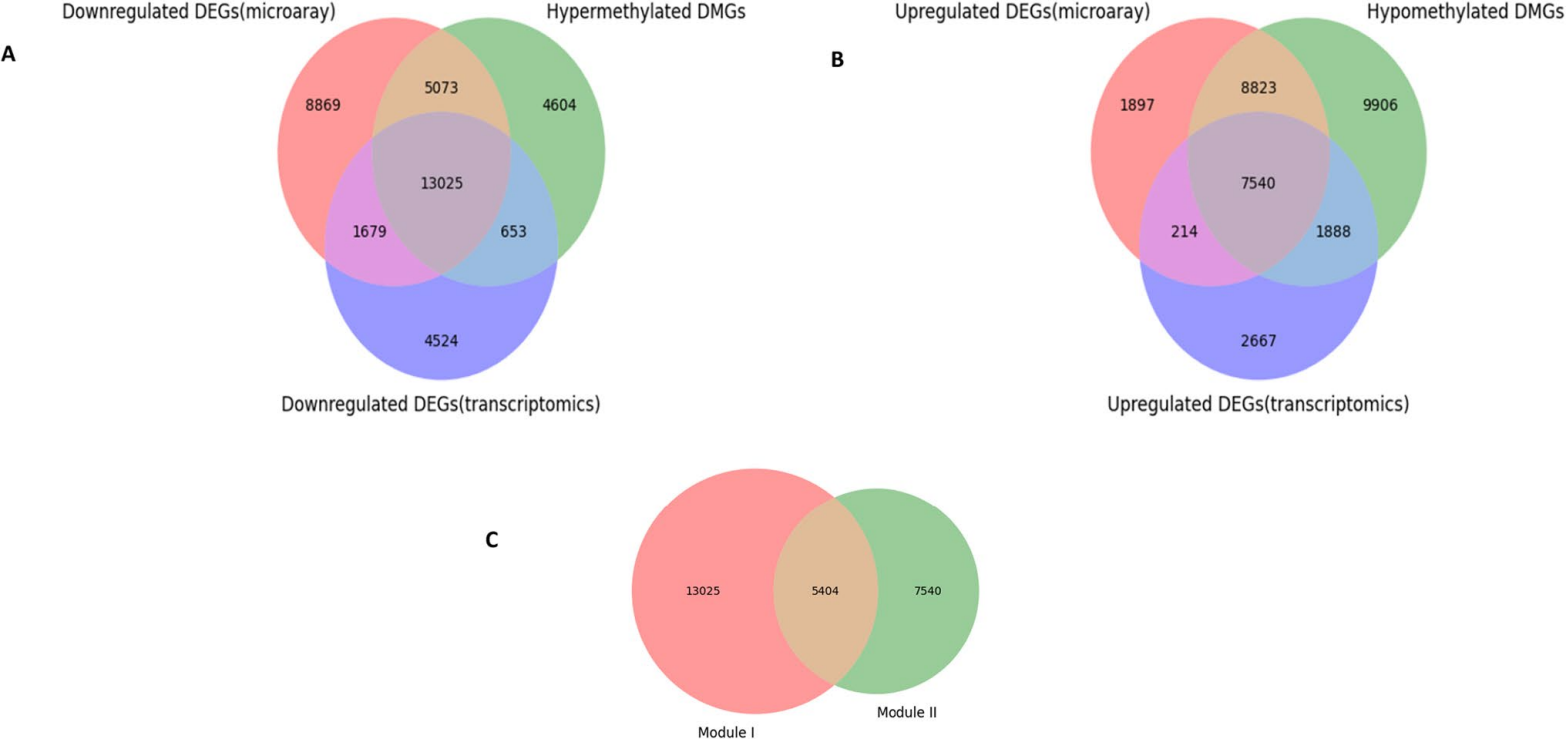
The differential common genes derived from the analysis of DNA methylation, RNA-seq and microarray gene expression data for colorectal cancer. **A** combination of the common Hypermethylated-downregulated genes (Module I); **B** combination of the common Hypomethylated-upregulated genes (Module II); **C** Calculation of overlapping genes between Module I and Module II

Matplotlib_venn python package was used for finding the common downregulated DEGs from microarray and RNA-seq dataset analysis along with hypermethylated DMGs, with the help of the Venn diagram, giving a count of 13025 genes (MODULE I) whereas the Venn diagram showing the combination of common upregulated DEGs from microarray and RNA-seq dataset analysis along with hypomethylated DMGs were constructed giving the count of 7540 genes (MODULE II). The visualization of the Venn diagram can be seen in (Fig. 2A and B). The downstream analysis of MeDEGs involves the removal of the overlapping differential genes with a count of 5404, functional enrichment analysis, and constructing a functional partners interaction network (Fig. 2C).

### Results for functional enrichment of MeDEGs

**Fig. 3:**
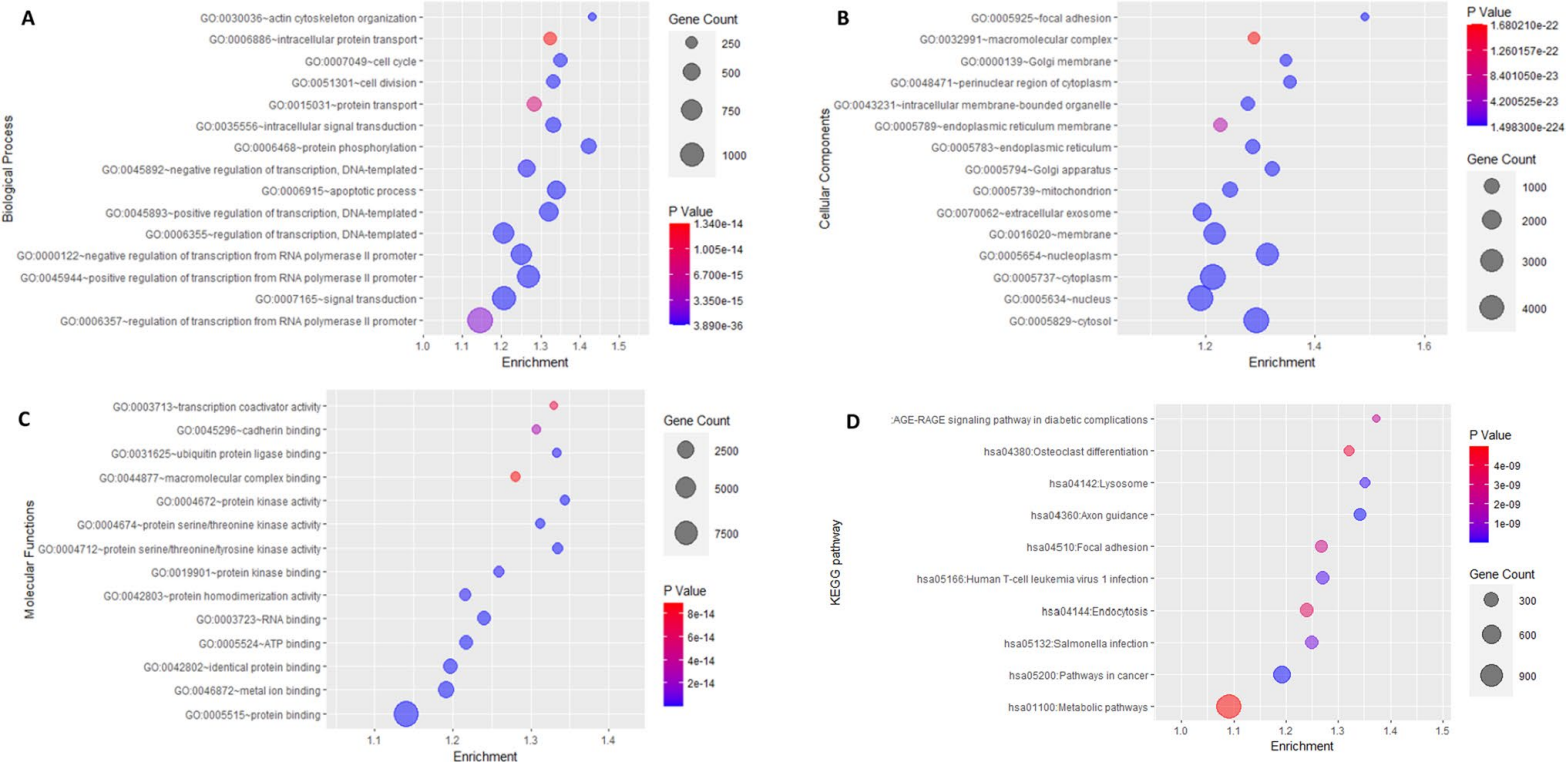
Dot-plot representing the functional enrichment of the different gene ontology terms and KEGG pathway of common Hypermethylated—downregulated genes (Module I). **A** Biological processes (BP) **B** Cellular Components (CC) **C** Molecular Functions (MF); **D** KEGG pathway

**Fig. 4:**
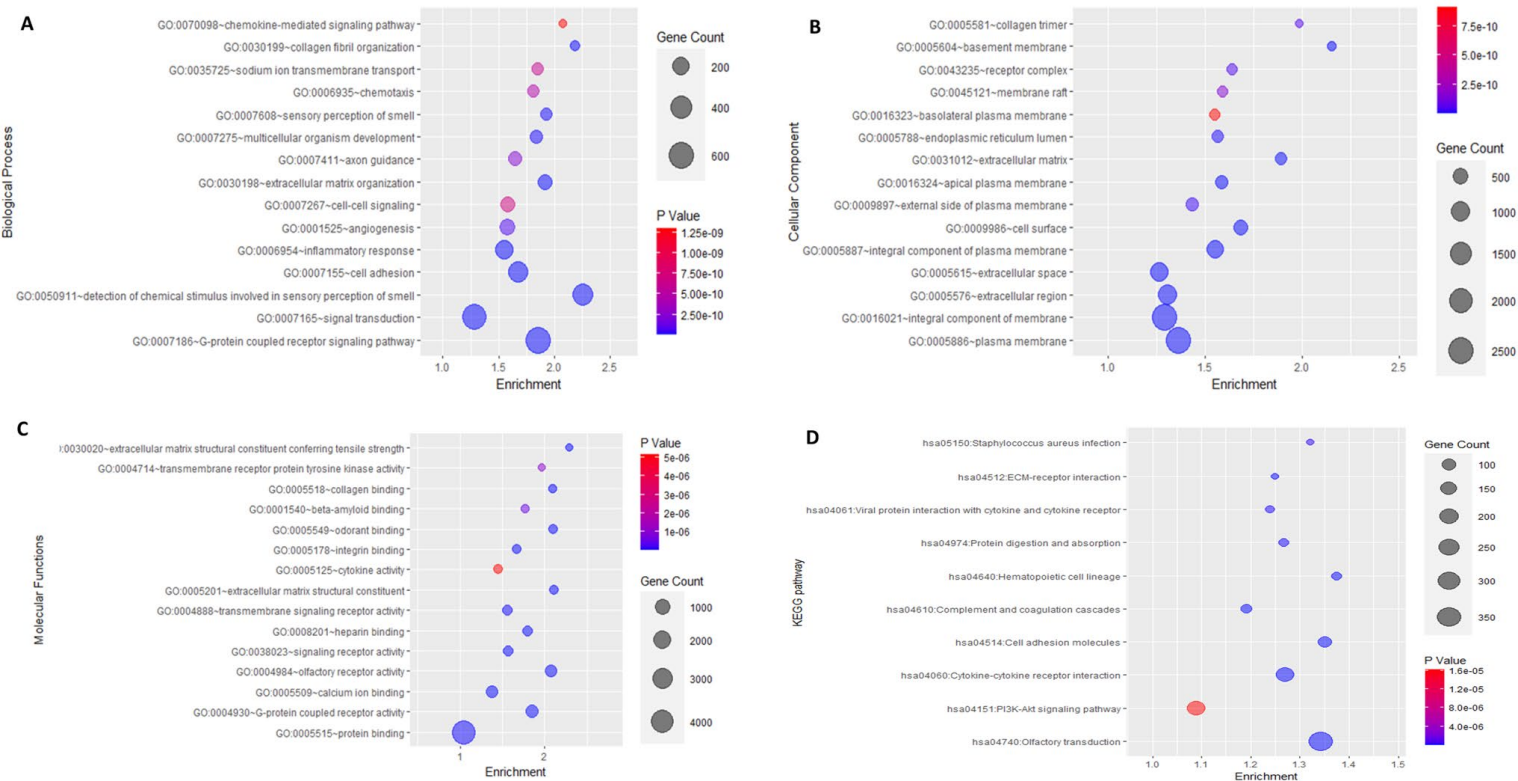
Dot-plot representing the functional enrichment of the different gene ontology terms and KEGG pathway of common Hypomethylated—upregulated genes (Module II). **A** Biological processes (BP); **B** Cellular Components (CC); **C** Molecular Functions (MF); **D** KEGG pathway

The functional enrichment analysis was performed for separate modules, and the top ten terms were selected for visualization with a threshold P < 0.05 in the BP, CC, and MF categories (Figs. 3 and 4 respectively). By filtering DEGs with a p-value threshold of 0.05, we identified significant gene ontology components, which are presented in Tables 4 and 5. For CC, genes show significant enrichment in the cytosol, nucleoplasm, cytoplasm, membrane, and integral component of the plasma membrane, whose disruption can trigger oncogenic signaling. Also, the results indicate that MeDEGs function in BP related to transcriptional regulation, protein phosphorylation pathways, signal transduction, and cell adhesion processes that regulate cellular functions like cell growth, differentiation, apoptosis, and signaling in healthy conditions. For MF, genes primarily focus on protein binding, metal ion binding, RNA, and ATP binding functions, G-protein coupled receptor activity, and other signaling receptor activity, causing the potential disruption in cellular mechanisms. The KEGG pathway analysis illustrated the genes concentrating on different cancer pathways, Axon guidance, Lysosome, Complement and coagulation cascades, Cell adhesion molecules, and Cytokine-cytokine receptor interaction pathways for both subtypes of differential genes (Figs. 3D and 4D) and Tables 4 and 5). This functional enrichment process became the criteria for selecting the MeDEGs which showed the specific nature of reactivity towards the initiation or persistence of CRC. The final number of selected enriched genes was 12,507.

**Table 4:** Gene ontology (GO) terms such as biological process, molecular functions, and KEGG pathways of DEGs that are associated with common hypermethylated- downregulated genes (Module I) from DAVID

S.No	Gene ontology ID	Description	Gene counts	P-value
Biological processes				
1	GO:0045944	Positive regulation of transcription from RNA polymerase II promoter	950	3.89104E-36
2	GO:0006468	Protein phosphorylation	385	3.53884E-33
3	GO:0045893	Positive regulation of transcription, DNA-templated	586	1.71069E-30
4	GO:0006915	Apoptotic process	505	3.20872E-29
5	GO:0000122	Negative regulation of transcription from RNA polymerase II promoter	772	3.5803E-26
6	GO:0007165	Signal transduction	960	5.26945E-23
7	GO:0035556	Intracellular signal transduction	374	5.9376E-21
8	GO:0007049	Cell cycle	304	5.16598E-19
9	GO:0030036	Actin cytoskeleton organization	203	1.3336E-18
10	GO:0006355	Regulation of transcription, DNA-templated	750	4.88731E-18
Cellular components				
1	GO:0005829	Cytosol	4328	1.4983E-224
2	GO:0005654	Nucleoplasm	3186	3.2985E-172
3	GO:0005737	Cytoplasm	4181	1.7891E-122
4	GO:0005634	Nucleus	4321	1.2007E-104
5	GO:0016020	Membrane	2671	7.35858E-72
6	GO:0005794	Golgi apparatus	901	5.56999E-45
7	GO:0005925	Focal adhesion	385	1.3099E-41
8	GO:0005783	Endoplasmic reticulum	899	8.91485E-37
9	GO:0048471	Perinuclear region of cytoplasm	607	5.79363E-36
10	GO:0005739	Mitochondrion	1087	3.84244E-34
Molecular functions				
1	GO:0005515	Protein binding	9141	1.6669E-278
2	GO:0046872	Metal ion binding	2059	2.20645E-48
3	GO:0003723	RNA binding	1165	1.49833E-39
4	GO:0005524	ATP binding	1188	1.03179E-33
5	GO:0042802	Identical protein binding	1317	1.39821E-31
6	GO:0004712	Protein serine/threonine/tyrosine kinase activity	376	3.18145E-23
7	GO:0004672	Protein kinase activity	327	1.88689E-21
8	GO:0004674	Protein serine/threonine kinase activity	335	1.68385E-18
9	GO:0031625	Ubiquitin protein ligase binding	266	2.33281E-16
10	GO:0042803	Protein homodimerization activity	577	2.64849E-16
KEGG pathway				
1	hsa04360	Axon guidance	167	1.08622E-13
2	hsa05200	Pathways in cancer	433	2.86373E-12
3	hsa04142	Lysosome	122	1.10812E-10
4	hsa05166	Human T-cell leukemia virus 1 infection	193	2.28798E-10
5	hsa05132	Salmonella infection	213	6.16182E-10
6	hsa04933	AGE-RAGE signaling pathway in diabetic complications	94	2.26269E-09
7	hsa04510	Focal adhesion	176	2.56898E-09
8	hsa04144	Endocytosis	212	3.4633E-09
9	hsa04380	Osteoclast differentiation	122	4.25827E-09
10	hsa01100	Metabolic pathways	1150	4.98925E-09

**Table 5:** Gene ontology (GO) terms such as biological process, molecular functions, and KEGG pathways of DEGs that are associated with common hypomethylated- upregulated genes (Module II) using DAVID

S.No	Gene ontology ID	Description	Gene counts	P-value
Biological processes				
1	GO:0007186	G-protein coupled receptor signaling pathway	617	8.10042E-81
2	GO:0050911	Detection of chemical stimulus involved in sensory perception of smell	336	6.83326E-77
3	GO:0007155	Cell adhesion	326	6.28148E-30
4	GO:0030198	Extracellular matrix organization	125	2.28275E-18
5	GO:0006954	Inflammatory response	233	2.86974E-16
6	GO:0007165	Signal transduction	580	1.88573E-14
7	GO:0030199	Collagen fibril organization	58	1.3581E-12
8	GO:0007608	Sensory perception of smell	79	3.84042E-12
9	GO:0007275	Multicellular organism development	91	4.9517E-12
10	GO:0001525	Angiogenesis	139	6.86396E-11
Cellular components				
1	GO:0005886	Plasma membrane	2523	1.4909E-108
2	GO:0016021	Integral component of membrane	2403	1.68493E-71
3	GO:0005887	Integral component of plasma membrane	767	8.58351E-53
4	GO:0009986	Cell surface	375	1.07241E-34
5	GO:0005576	Extracellular region	982	1.12237E-27
6	GO:0031012	Extracellular matrix	166	5.03139E-23
7	GO:0005615	Extracellular space	852	9.3808E-19
8	GO:0016324	Apical plasma membrane	219	2.44361E-16
9	GO:0005604	basement membrane	70	3.0831E-14
10	GO:0005788	Endoplasmic reticulum lumen	165	4.96104E-12
Molecular functions				
1	GO:0004930	G-protein coupled receptor activity	524	2.30875E-71
2	GO:0004984	Olfactory receptor activity	336	2.41872E-63
3	GO:0005201	Extracellular matrix structural constituent	105	4.74182E-21
4	GO:0005549	Odorant binding	95	5.0691E-19
5	GO:0005509	Calcium ion binding	374	5.05766E-15
6	GO:0008201	Heparin binding	115	2.47888E-14
7	GO:0005518	Collagen binding	51	2.4817E-10
8	GO:0038023	Signaling receptor activity	131	4.56978E-10
9	GO:0005178	Integrin binding	96	9.56828E-10
10	GO:0030020	Extracellular matrix structural constituent conferring tensile strength	37	1.57393E-09
KEGG pathway				
1	hsa04740	Olfactory transduction	351	7.65E-66
2	hsa04610	Complement and coagulation cascades	75	2.81855E-18
3	hsa04514	Cell adhesion molecules	105	1.23276E-10
4	hsa04060	Cytokine-cytokine receptor interaction	174	4.71569E-10
5	hsa04512	ECM-receptor interaction	65	2.18814E-09
6	hsa04640	Hematopoietic cell lineage	70	4.52493E-09
7	hsa04974	Protein digestion and absorption	70	5.7382E-08
8	hsa04061	Viral protein interaction with cytokine and cytokine receptor	68	8.85104E-08
9	hsa05150	Staphylococcus aureus infection	65	2.27779E-07
10	hsa04151	PI3K-Akt signaling pathway	186	1.62154E-05

### Machine learning analysis

#### Class imbalance and feature selection

The obtained dataset comprised highly imbalanced data containing the normal class with 51 samples and 648 tumor samples, for transcriptomics profiling data. Similarly, the methylation datasets (containing 27 K and 450 K) showed an imbalance with 742 tumor samples and 119 normal samples. The tumor and normal sample percentages in the imbalanced data are 92.68% and 7.31% respectively, as shown in *Supplementary Fig. 4A and 4B.* Before applying feature selection methods, we performed several undersampling and oversampling techniques such as RandomUnderSampler, TomekLinks, RandomOversampler, and SMOTE. To enhance model performance, we prioritized oversampling methods like SMOTE, which adds data to the minority class to reduce information loss. Moreover, the high dimensionality of the TCGA datasets complicates classification, making the LASSO algorithm essential for selecting the most relevant genes for CRC prediction. Hence, LASSO provided a total of 1421 features from both transcriptomics and methylation datasets with 5000 iterations and alpha of 0.0013.

#### Machine learning classifier

The classification reports generated for the mentioned classification models (KNN, ANN, RF) built on TCGA datasets, show a range between 99 and 100% accuracy. The classification report generated for the Random Forest and KNN classification model is tabulated in Table 6. The anticipated classification accuracy for the ANN, KNN, and RF models stands at 100%, 100%, and 99.99% for transcriptomics data, respectively. Similarly, for the 450 k methylation data platform, the accuracies are 100%, 99.85%, and 100%, while for the 27 k data platform, all models achieve 100% accuracy. Learning plots display the ANN model’s improving performance over time, ensuring effective training without overfitting or underfitting *(refer Supplementary Fig. 5).* The models achieved a 100% accuracy rate when their predictions were evaluated using the test data (unseen). Moreover, the AUC value of the classification models achieves 1.00, indicating their ability to perfectly differentiate between positive and negative classes without any errors (*Supplementary Fig. 6*).

**Table 6:** The classification report of the generated random forest and KNN classification model for both transcriptomics and DNA methylation CRC dataset

	Transcriptomics TCGA-CRC datasets analysis							
	Random forest				KNN model			
	Precision	Recall	F1-score	Support	Precision	Recall	F1-score	Support
Normal	1.00	0.98	0.99	52	0.99	1.00	1.00	53
Tumour	0.98	1.00	0.99	58	1.00	0.99	1.00	57
Accuracy			0.99	110			1.00	110
Macro avg	0.99	0.99	0.99	110	1.00	1.00	1.00	110
weighted avg	0.99	0.99	0.99	110	1.00	1.00	1.00	110
	DNA methylation TCGA-CRC datasets analysis (Illumina human methylation 450 k platform)							
	Random forest				KNN model			
	Precision	Recall	F1-score	Support	Precision	Recall	F1-score	Support
Normal	1.00	1.00	1.00	38	1.00	1.00	1.00	38
Tumour	1.00	1.00	1.00	32	1.00	1.00	1.00	32
Accuracy			1.00	70				70
Macro avg	1.00	1.00	1.00	70	1.00	1.00	1.00	70
weighted avg	1.00	1.00	1.00	70	1.00	1.00	1.00	70
	DNA methylation TCGA-CRC datasets analysis (Illumina human methylation 27 k platform)							
	Random forest				KNN model			
	Precision	Recall	F1-score	Support	Precision	Recall	F1-score	Support
Normal	1.00	1.00	1.00	28	1.00	1.00	1.00	28
Tumour	1.00	1.00	1.00	29	1.00	1.00	1.00	29
Accuracy			1.00	57			1.00	57
Macro avg	1.00	1.00	1.00	57	1.00	1.00	1.00	57
weighted avg	1.00	1.00	1.00	57	1.00	1.00	1.00	57

### Results for evaluation of the functional interactions, Gene interacting partner network construction, and selection of Hub genes

**Fig. 5:**
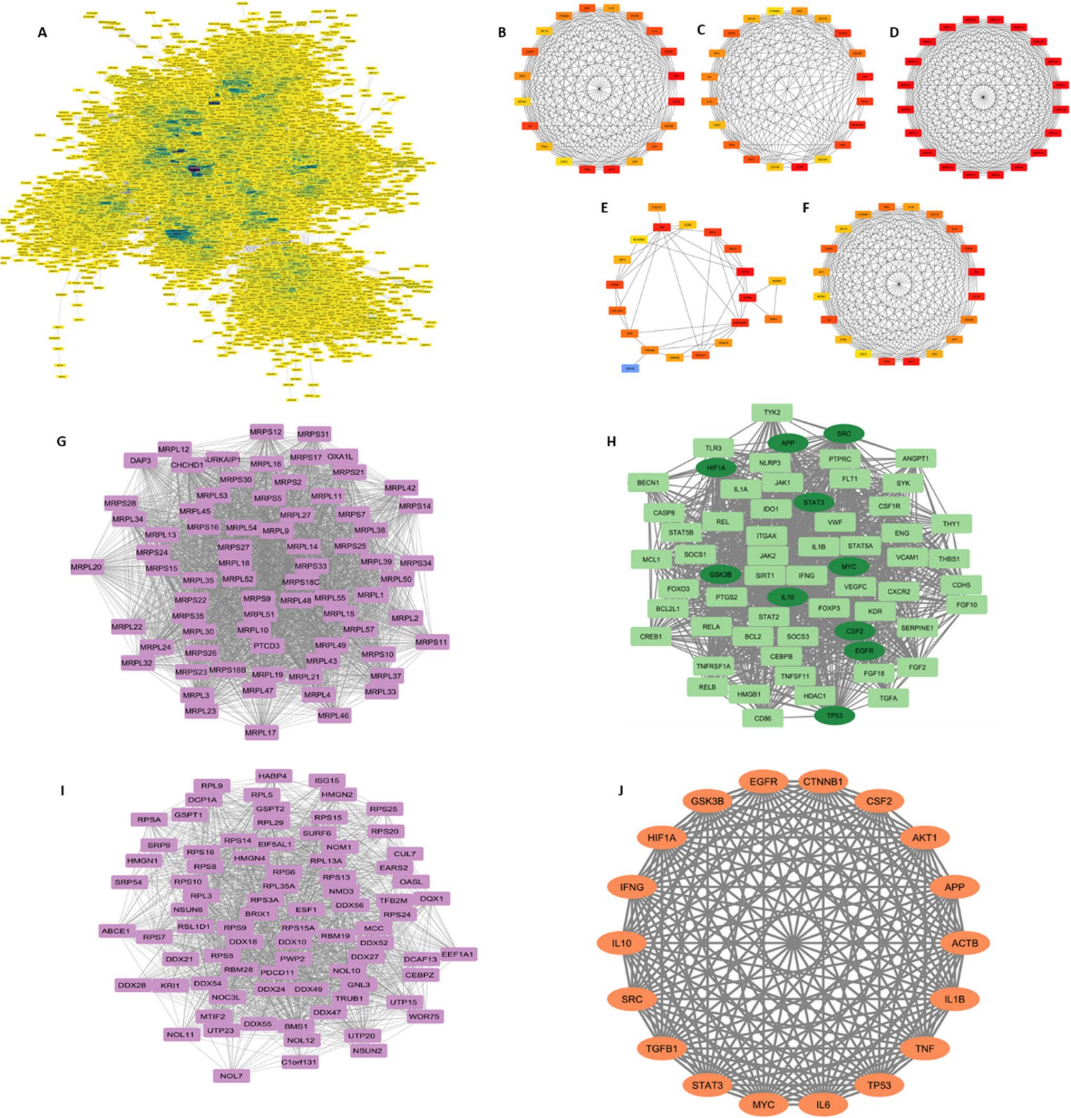
A Gene interacting partner network of the total enriched genes. The colour depiction in the network is based on the continuous mapping with the change in colour indicating the degree of interaction. Estimation of Hub genes using Cytohubba plugin in Cytoscape software. The representation of five different metrics are as follows: **B** DEGREE; **C** closeness; **D** MCC; **E** betweenness; **F** MNC. The rectangle shaped genes are the correlated genes found from the PPI network using MCODE; **G** MCODE CLUSTER 1 (score = 74.00). **I** MCODE CLUSTER 2 (score = 43.38), respectively. **H** The diamond shaped genes are the hub genes whereas the rectangle shaped genes are the corelated genes. MCODE CLUSTER 3(score = 39.129); **J** The derived Hub genes from statistical approach

**Fig. 6:**
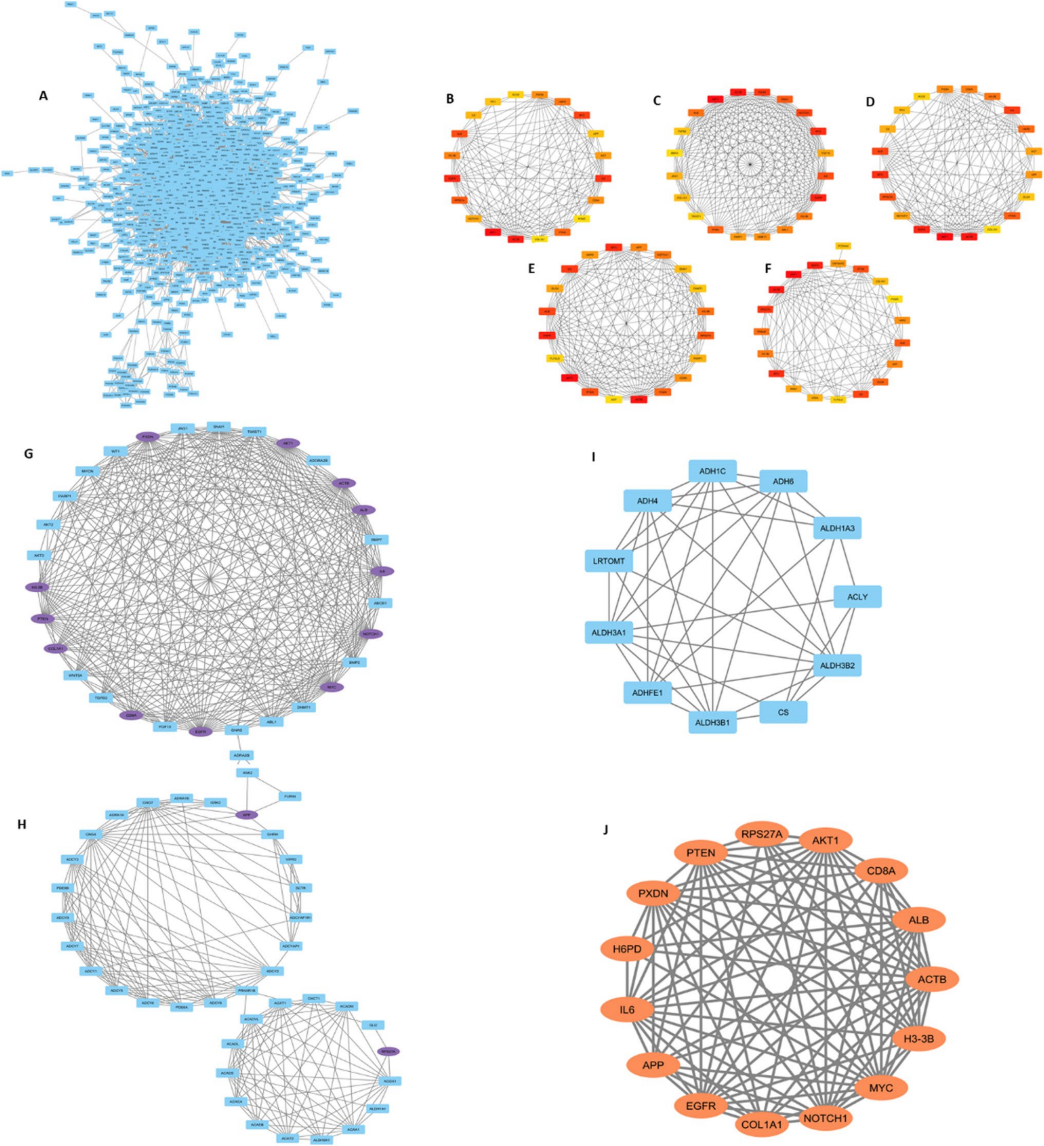
**A** Gene interacting partner network of the total features. Estimation of Hub genes using Cytohubba plugin in Cytoscape software. The representation of five different metrics are as follows: **B** MNC; **C** MCC; **D** Degree; **E** Closeness; and **F** Betweenness; The rectangle shaped genes are the correlated genes found from the PPI network using MCODE. **G** MCODE CLUSTER 1 (score = 17.56). **H** MCODE CLUSTER 2 (score = 9.421), respectively. **I** The diamond shaped genes are the hub genes whereas the rectangle shaped genes are the corelated genes. MCODE CLUSTER 3(score = 7.8); **J** The derived Hub genes from Machine learning approach

**Fig. 7:**
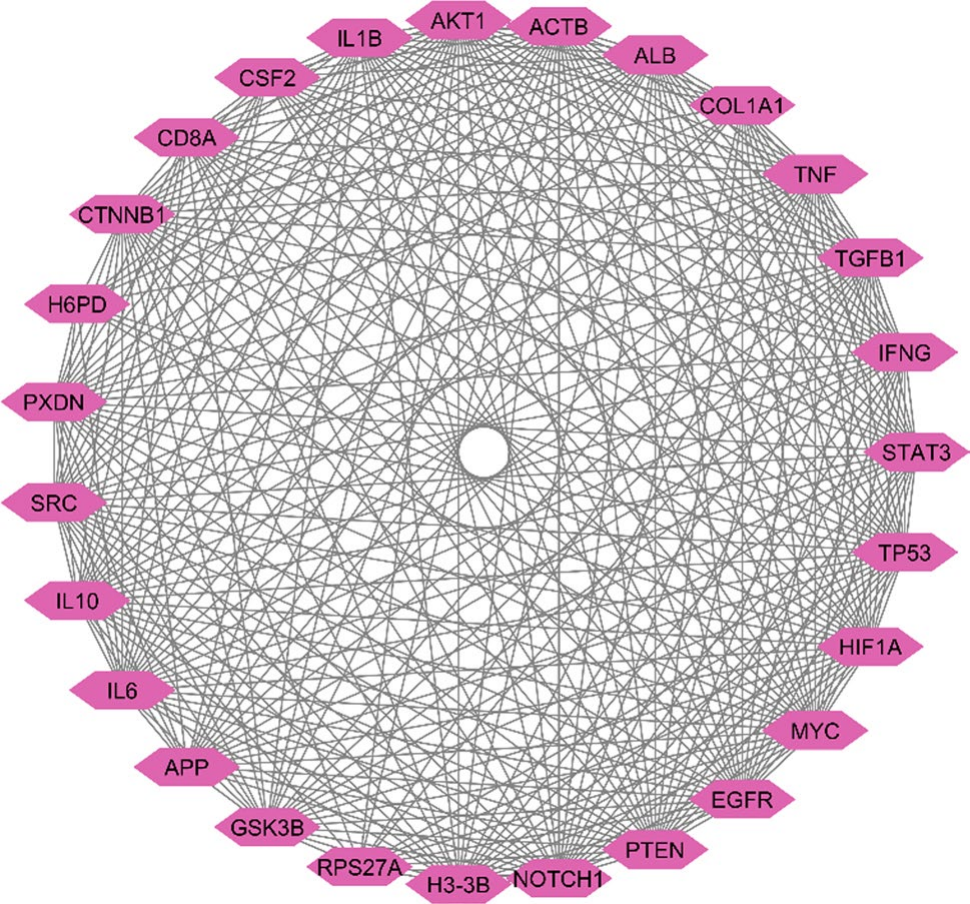
Visualization of the Hub genes derived from both statistical and machine learning approach from GEO and TCGA-GDC datasets

The Gene-interacting partner network of enriched genes was constructed using the STRING database and was displayed by using Cytoscape (Fig. 5A) containing 1129 nodes and 9205 edges with an average node degree of 16.29, and an average local clustering coefficient of 0.32. In this context, the edges symbolize the interactions between the genes, while the nodes represent the genes themselves. The Cytohubba plugin facilitated the generation of the top 20 hub genes using different global and local methods as depicted in (Fig. 5C–F). Five different algorithms, namely Degree (Fig. 5B); closeness (Fig. 5C); MCC (Fig. 5D); betweenness (Fig. 5E); and MNC (Fig. 5F) were utilized to derive the hub genes (Table 7) by analyzing the enriched genes that are present in three or more than three scoring algorithms. Those are AKT1, MYC, CTNNB1, STAT3, IL6, SRC, TP53, CSF2, TNF, EGFR, APP, GSK3B, ACTB, HIF1A, TGFB1, and IL10. Through MCODE analysis, we chose the three most important modules (MCODE score = 74.00, 43.38, and 39.129) (refer Fig. 5G, H, I) The MCODE analysis clustered group genes influence ribosomal protein synthesis in cancer cells, linked to dysregulation of key cancer-related proteins like c-Myc, mTOR, p53, pRB, and PTEN, and irregular expression of mitochondrial translational factors [32]. The additional two genes (IL-1β and IFNG) can be also considered as the Biomarker genes along with the hub genes (derived from Cytohubba) for CRC, resulting in a complete hub gene network (refer Fig. 5J). Similarly, 1421 features obtained from the ML approach were subjected to PPI network formation (Fig. 6A), Cytohubba, and MCODE analysis (Fig. 6B–I) for identifying 15 Hub genes (Fig. 6J). Interestingly, the analysis revealed that the MCODE application yielded no new genes compared to Cytohubba. Furthermore, the Biomarker genes derived from the ML analysis are as follows: IL6, H3-3B, AKT1, MYC, PTEN, ALB, EGFR, ACTB, APP, CD8A, H6PD, RPS27A, COL1A1, NOTCH1, and PXDN (refer Table 8). Individual gene functions were derived from the GeneCards database (GeneCards—Human Genes | Gene Database | Gene Search). Hence, by treating the overlapping biomarker genes from both approaches as a single entity, we have identified 27 hub genes for subsequent downstream analysis. The visualization of the same can be seen in Fig. 7 and further information regarding the hub genes can be detailed in *Supplementary Table 2*.

**Table 7:** The list of enriched genes showing highest interaction according to different topological algorithms (MNC, MCC, Degree, Closeness, Betweenness, using Cytohubba

S.No	MCC	MNC	Betweenness	Degree	Closeness
1	MRPS17	NFKB1	SCARB2	NFKB1	APP
2	MRPL55	APP	HSPA9	APP	IL10
3	MRPS28	IL10	HDAC4	IL10	AKT1
4	MRPL14	AKT1	APP	AKT1	MYC
5	MRPS26	MYC	RPL5	MYC	CTNNB1
6	MRPL38	IFNG	FEN1	IFNG	STAT3
7	MRPL39	CTNNB1	TSG101	CTNNB1	TP53
8	MRPL42	STAT3	EIF4A1	STAT3	CSF2
9	MRPL57	TP53	RPL4	TP53	EGFR
10	MRPS27	CSF2	TNF	CSF2	RPL4
11	MRPL48	EGFR	COL18A1	EGFR	TNF
12	CHCHD1	TNF	HSP90AB1	TNF	CSF1R
13	MRPS23	IL1B	TOP2A	IL1B	HSP90AB1
14	MRPL54	IL6	SPI1	IL6	BECN1
15	MRPS33	SRC	YWHAE	SRC	IL6
16	MRPL35	CD4	UBA52	CD4	SRC
17	MRPL30	GSK3B	CD86	GSK3B	GSK3B
18	MRPL50	ACTB	ACTB	ACTB	ACTB
19	MRPL18	HIF1A	MUS81	HIF1A	HIF1A
20	MRPL52	TGFB1	TGFB1	TGFB1	TGFB1

**Table 8:** The list of feature genes showing highest interaction according to different topological algorithms (MNC, MCC, Degree, Closeness, Betweenness, using Cytohubba

S.No	MCC	MNC	Betweenness	Degree	Closeness
1	IL6	APP	APP	APP	APP
2	H3-3B	IL6	IL6	IL6	IL6
3	FGF10	H3-3B	H3-3B	H3-3B	H3-3B
4	AKT1	CD8A	CD8A	CD8A	CD8A
5	MYC	TPI1	PCDHA4	TPI1	AKT1
6	TGFB2	ACO2	AKT1	ACO2	MYC
7	NOTCH1	AKT1	MYC	AKT1	H6PD
8	COL1A1	MYC	H6PD	MYC	RPS27A
9	DNMT1	H6PD	RPS27A	H6PD	NOTCH1
10	BMP2	RPS27A	COL1A1	RPS27A	DNMT1
11	ABL1	NOTCH1	POMC	NOTCH1	FLT3LG
12	PTEN	COL1A1	FLT3LG	COL1A1	DLG4
13	TWIST1	CS	DLG4	DLG4	PTEN
14	PARP1	PTEN	PTEN	CS	AGT
15	PXDN	AGT	CNTNAP2	PTEN	PARP1
16	ALB	PXDN	TRIM28	AGT	PXDN
17	SNAI1	ALB	ANK2	PXDN	ALB
18	EGFR	EGFR	ALB	ALB	SNAI1
19	ACTB	GNG7	EGFR	EGFR	EGFR
20	JAG1	ACTB	ACTB	ACTB	ACTB

### Regulatory network analysis of Hub genes

#### MicroRNAs network of hub genes

**Fig. 8:**
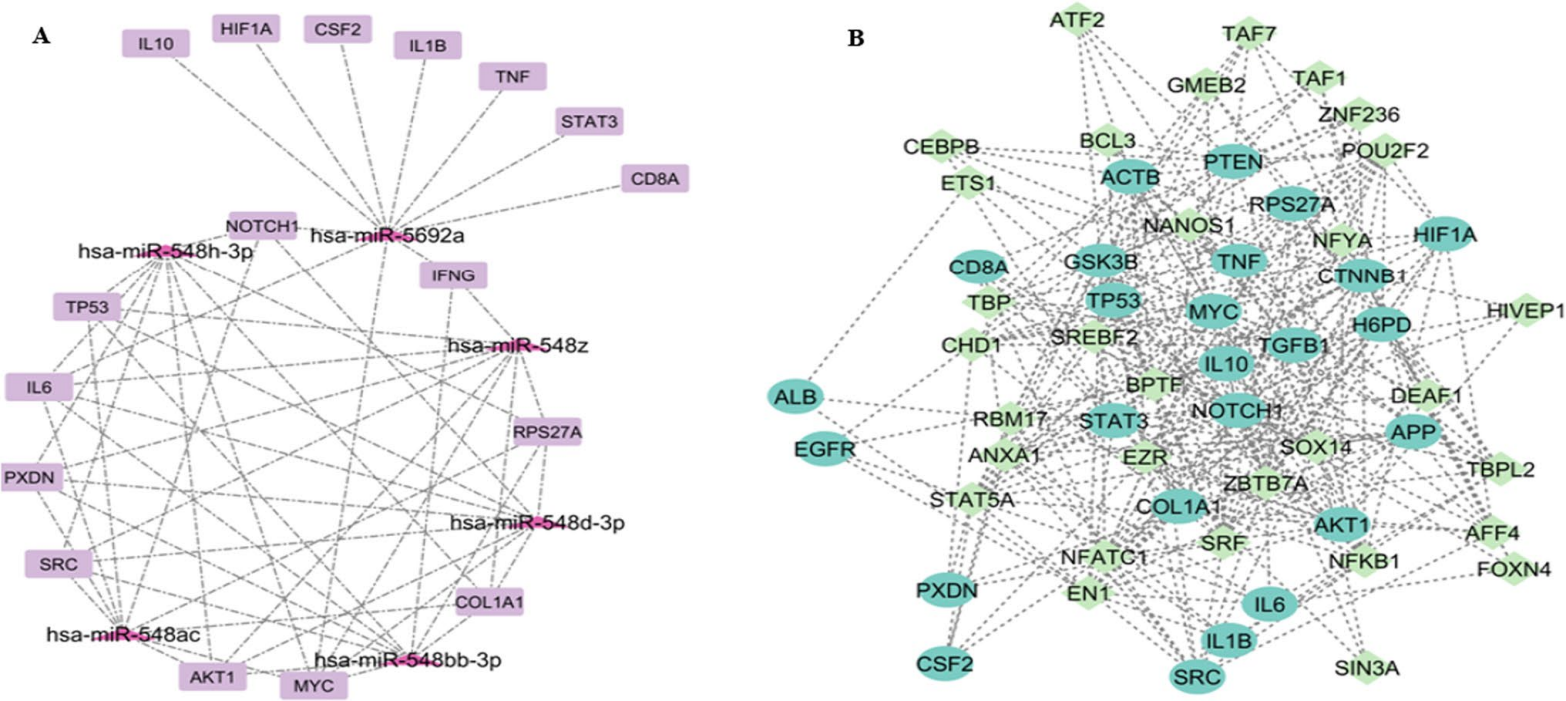
**A** This is the network showcasing the high frequency of interacting miRNA and the respective associated hub genes. The pink triangles are miRNAs and the purple rectangular entities are the hub genes in the network with 43 nodes and 74 edges. **B** This figure depicts the regulatory network as the Transcription factor and hub genes giving 56 nodes and 278 edges where the aqua blue eclipse shaped objects in the network denote hub genes and the diamond green shaped objects denote TFs. Few of the hub genes that are themselves transcriptional factors are MYC, CTNNB1, STAT3, TP53, and HIF1A

To identify the top-ranking micro-RNA (miRNA) serving as crucial post-transcriptional regulators of Hub genes, we constructed a Hub genes-miRNAs interaction network with 1616 nodes and 3127 edges. Through this network, we selected key regulators showing the highest degree of association with the majority of hub genes (17 genes) are hsa-miR-548ac, hsa-miR-548 h-3p, hsa-miR-548bb-3p, hsa-miR-548d-3p, hsa-miR-548z, hsa-miR-5692a. Figure 8A analyzed the selected miRNAs and their respective associated hub genes network with 23 nodes and 56 edges.

##### Transcription factor network with hub genes

Also, we constructed hub genes and transcription factors interaction networks to derive top-ranking TFs as the key transcriptional regulators of hub genes. A total of 46 TFs were derived by the iRegulon and Fig. 8B shows the constructed network (56 nodes and 278 edges) where the blue color eclipse-shaped objects in the network denote TFs and the diamond-green-shaped objects denote hub genes. We selected the top 5 key TFs (BPTF, SRF, STAT5A, ZBTB7A, and NFKB1) as the vital transcriptional regulators with a high degree of interaction within the hub genes-TF interaction network.

### Cell-specific expression patterns of hub genes in colorectal cancer using scRNA-seq analysis

**Fig. 9:**
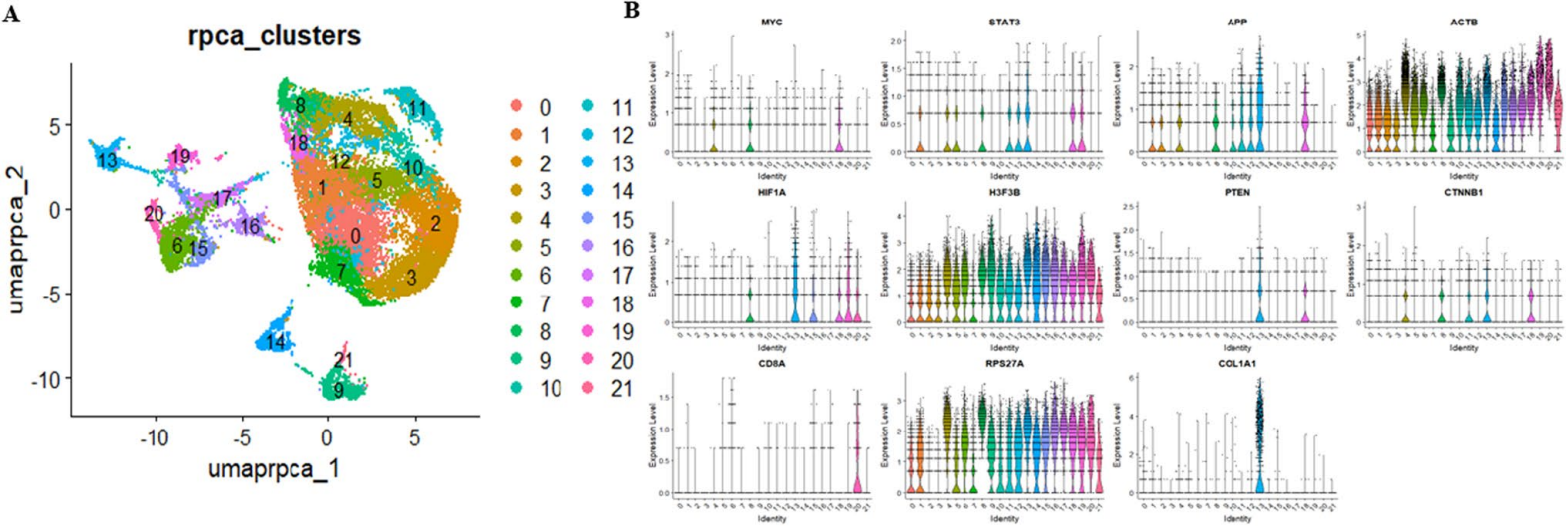
Single-cell RNA sequence analyses. **A** UMAP plot representation of cells from CRC and normal samples and clusters; **B** Violin plot determining the expression of few hub genes

This analysis enabled us to identify and characterize distinct cell populations within CRC. Subsequently, after preprocessing the data, the cells derived from five patients (both control and diseased) samples were clustered into 22 distinct clusters via the UMAP algorithm (Fig. 9A). FindAllMarkers() defines clusters using differential expression (DE) giving a list of DEGs. Later, we examined the cell-specific expression patterns of all hub genes among which only 11 hub genes showed good expression values. The violin plots illustrated that ACTB, RPS27A, and H3F3B were highly expressed followed by expression of STAT3, APP, HIF1A, and CTNNB1 genes (Fig. 9B). Validation of almost 20 hub genes (excluding IL6, CSF2, IL10, ALB, H6PD, and PXDN) as marker genes were seen within the list of DEGs derived from separate scRNA-seq count datasets underscores their biological relevance, indicating their involvement not only in bulk tissue samples but also in specific cell types or subpopulations. These validated hub genes represent promising biomarker candidates deserving further investigation.

### The validation of promoter methylation and expression analysis

**Fig. 10:**
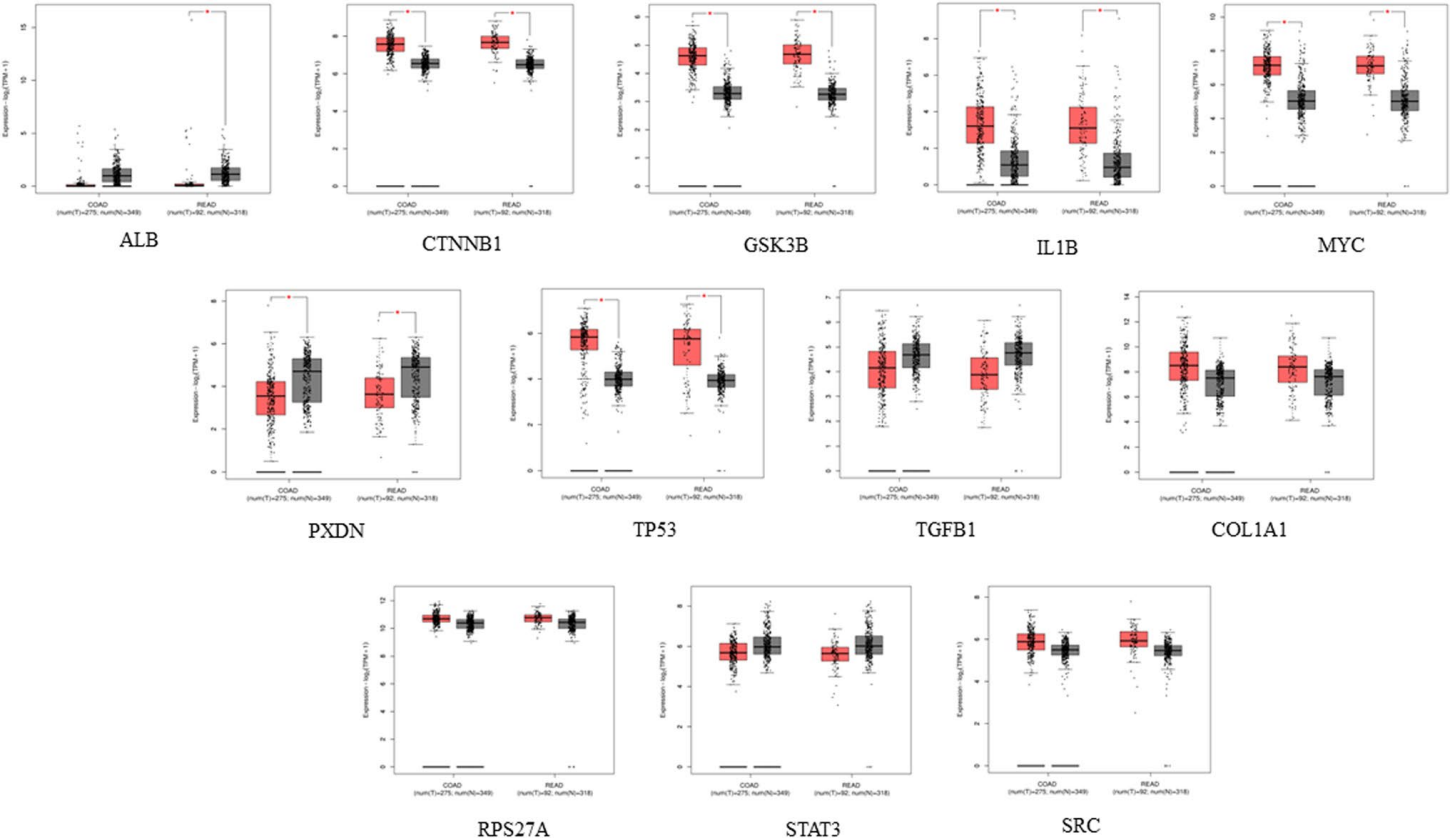
Differential gene expression analysis of tumor and normal tissues in GEPIA. *ALB, CTNNB1, GSK3B, IL1B, MYC, PXDN, TP53, TGFB1, COLA1, RPS27A, STAT3, and SRC* are the genes that show effective expression in TCGA-COAD and TCGA-READ patients samples*.* *P < 0.05

**Fig. 11:**
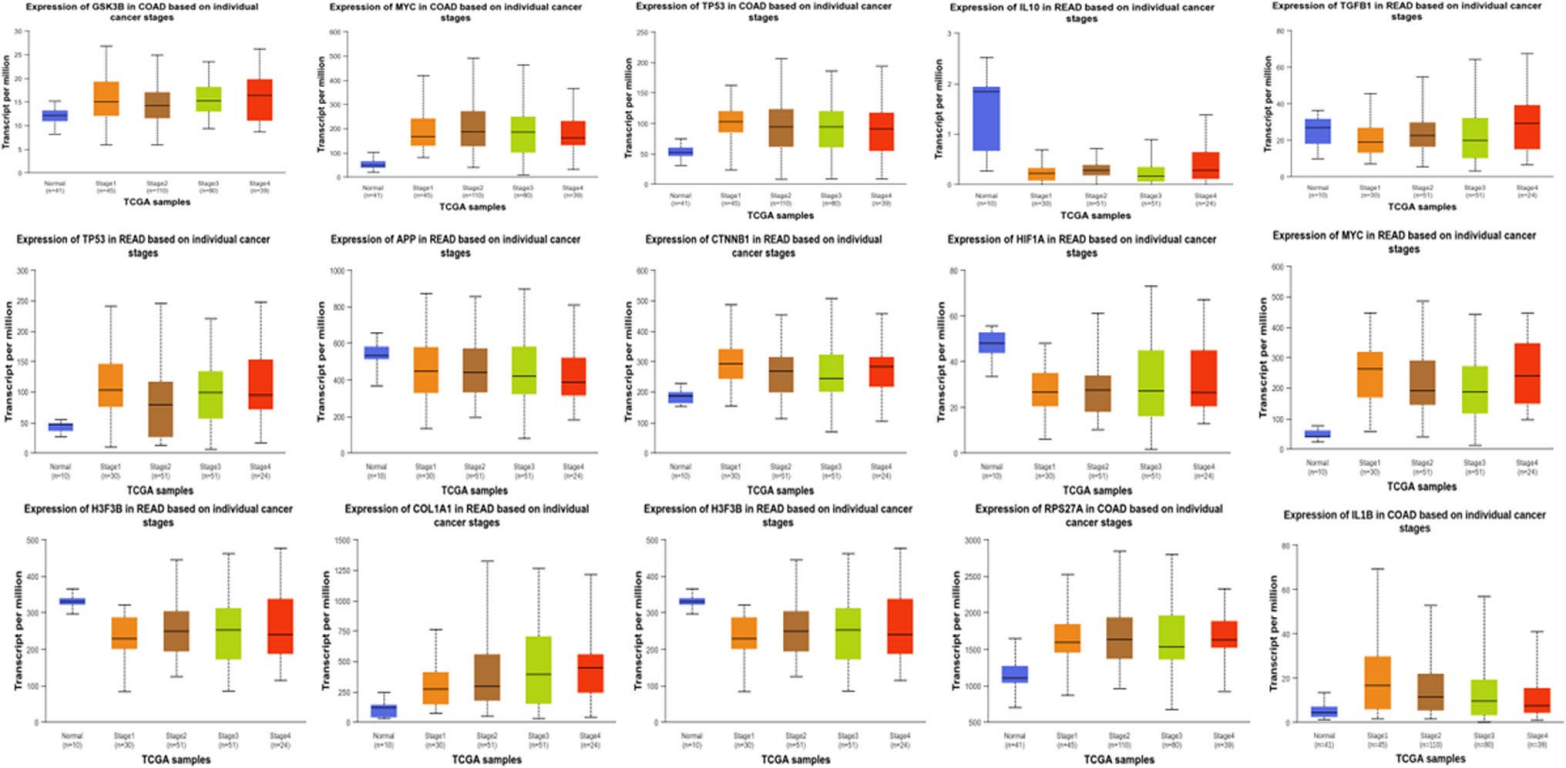
Expression profile of CTNNB1, GSK3B, MYC, TP53, APP, HIF1A, IL1B, RPS27A, COL1A1, IL10, TGFB1 and H3-3B in colon cancer and rectum adenocarcinoma patient’s subgroups, stratified based on stage criteria (UALCAN)

The methylation levels of all hub genes were found to be increased in patients’ samples, as all the genes showed p-values lower than 0.001. According to our analysis, the validation of the promoter methylation of hub genes is analyzed from TCGA Illumina bead chip data using UALCAN, which shows an inverse relation with gene expression profile, stating the significant statistical significance in COAD patients (AKT1, EGFR, IFNG, PXDN, H6PD, MYC, GSK3B, APP, NOTCH1, RPS27A) and READ patients (IFNG, PXDN, NOTCH1, GSK3B, TP53, MYC, PTEN, TGFB1, H6PD, IL-1β). The genes validating hypomethylation are AKT1, CTNNB1, IL6, SRC, CSF2, TNF, ACTB, HIF1A, IL10, IL-1β, IFNG, PTEN, ALB, RPS27A, COL1A1, MYC, APP whereas the hypermethylated genes are STAT3, TP53, EGFR, GSK3B, TGFB1, CD8A, H6PD, NOTCH1. Other gene expression analysis involves the results showing that upregulated differential expression *CTNNB1, GSK3B, IL-1β, MYC, PXDN, TP53, COL1A1, RPS27A,* and *SRC* genes out of all the derived hub genes in cancer tissues (p < 0.05; Fig. 10). Additionally, the expressions of CTNNB1, GSK3B, MYC, TP53, and RPS27A differed significantly between stage IV colon cancer and normal (p < 0.05; Fig. 11), while that of *APP, CTNNB1, HIF1A, MYC, TP53, COL1A1, and H3-3B* differed significantly between stage IV rectum adenocarcinoma and normal tissues.

### Prognostic analysis and correlational analysis with immune cells

**Fig. 12:**
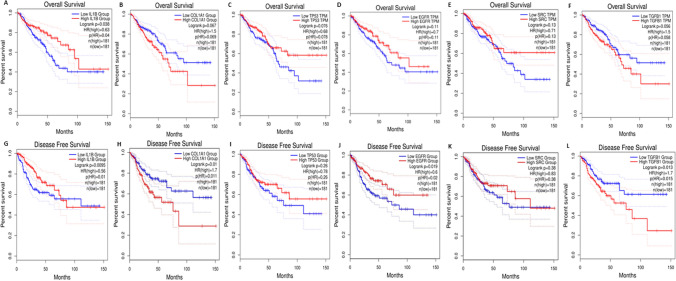
Kaplan–Meier survival curve of colon and rectal adenocarcinoma (COAD and READ) cohort with high and low expression of genes ILB1, COL1A1, TP53, EGFR, SRC, TGFB1 were analyzed by the GEPIA database (**A–L**). **A, G** High IL-1β expression was related to poorer OS and DFS in READ and COAD cohorts. (**B, H, C, I, F**, and **L**) shows the high expression of COL1A1, TGFB1, and TP53 are related to poor OS and DFS. (**D, E, J**, and **K**) reflects that the expression of EGFR and SRC genes are related to better OS and DFS in READ and COAD cohorts. OS, overall survival; DFS, Disease-free survival

**Fig. 13:**
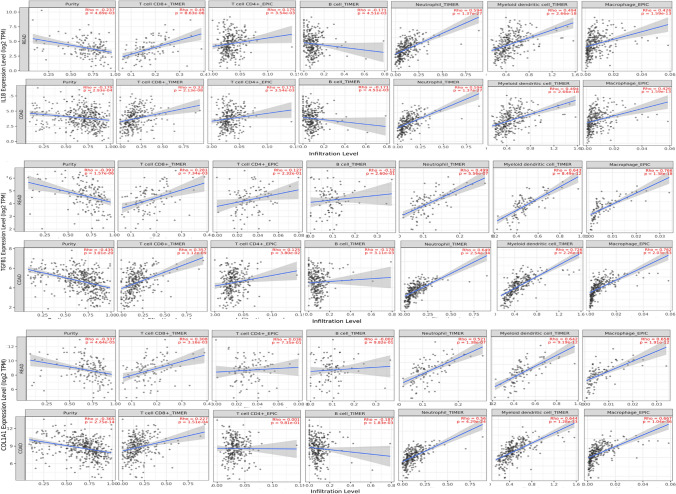
Correlation of hub genes showing prognostic expression with immune infiltration in CRC. Correlation of IL-1B, TGFB1, COL1A1 gene expression with infiltration levels of CD8 + T cell, CD4 + T cell, B cell, neutrophil, macrophage, and dendritic cells in CRC available at TIMER database. TILs: tumor-infiltrating lymphocytes; TIMER: Tumor Immune Estimation Resource. Color images are available online

Through the previous analysis, we identified ten genes as the candidate diagnostic biomarker genes which can be further subjected to prognostic and correlational analysis. These genes are CTNNB1, GSK3B, IL-1β, MYC, PXDN, TP53, EGFR, SRC, COL1A1 and TGBF1. The prognostic value of all biomarker expressions resulted in high expression of IL-1β that can be associated with the poorer overall survival of patients and DFS (Disease-free survival) in COAD and READ (n = 362, OS: HR = 0.63, P = 0.038; DFS: HR = 0.56, P = 0.0095). Figure 12A–L shows the Kaplan–Meier survival curve of colon and rectal adenocarcinoma with high and low IL-1β, COL1A1, TP53, EGFR, SRC, TGFB1 expression analyzed by the GEPIA database. The results of the survival curve analysis indicated that the IL-1β gene may serve as a better prognostic biomarker, evidenced by the smallest p-value indicating a stronger association with patient survival. For correlation analysis, our observations revealed that most of these prognosis-related genes displayed positive correlations (rho values) with the levels of infiltration of various immune cell types, while showing negative associations with tumor purity (see Fig. 13). Particularly noteworthy, IL-1β, TGFB1, and COL1A1 genes exhibited remarkable positive correlations with the levels of infiltration of the six immune cell types.

## Discussion

The detection of early-stage CRC has an admirable prognosis compared to advanced-stage, where patients often face challenges due to frequent recurrences and the incurable nature of the disease [33]. The increasing mortality rates highlight the importance of monitoring biomarker studies for screening, risk assessment, staging, therapy selection, and tracking recurrent disease [34]. The development of novel biomarkers for early detection and prognosis is crucial for refining personalized treatment strategies in clinical practice, despite ongoing challenges.

The present study aims to systematically utilize a dual approach involving bioinformatics and machine learning analysis to elucidate the key effects of candidate genes, which serve as diagnostic and prognostic biomarkers in CRC. The 27 hub genes identified in this study are implicated in key cancer signaling pathways, including Wnt, cAMP, VEGF, PI3K-Akt, and Jak-STAT. Previous studies have shown that deregulation in the PI3K/Akt and Wnt pathways, involving genes like AKT and MYC, is linked to CRC progression [35, 36]. Additionally, alterations in EGFR and TGFβ pathways promote tumor development, while STAT3 and p53 enhance cell proliferation and migration [37, 38]. Moreover, the derived regulatory elements along with Hub genes are implicated in modulating the cancer signaling pathways and other disease mechanisms (*refer Supplementary Table 3*). Notably, we establish that (i) IL-1β can be a better diagnostic and prognostic biomarker and predicts immune cell infiltration for CRC; (ii) We propose a cyclical regulatory mechanism involving hub genes and regulatory elements that mutually activate and regulate the PI3K/AKT signaling pathway in CRC; (iii) IL-1β, IL-6 and TNF-α expression is associated with inflammatory cell infiltration which can exhibit the progression of CRC.

**Fig. 14:**
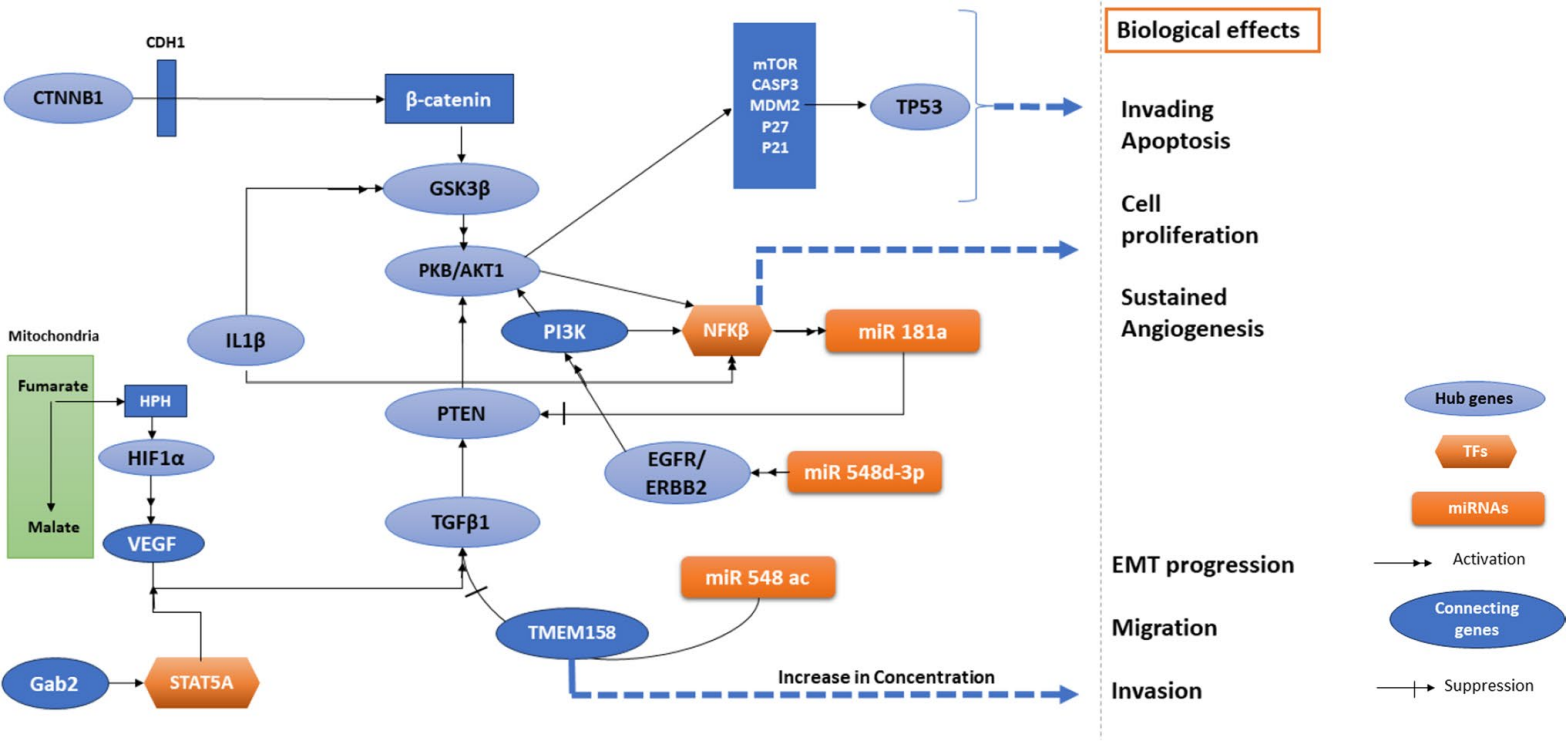
The visualization of complete signaling and channelling pathways occurring in colorectal cancer

**Fig. 15:**
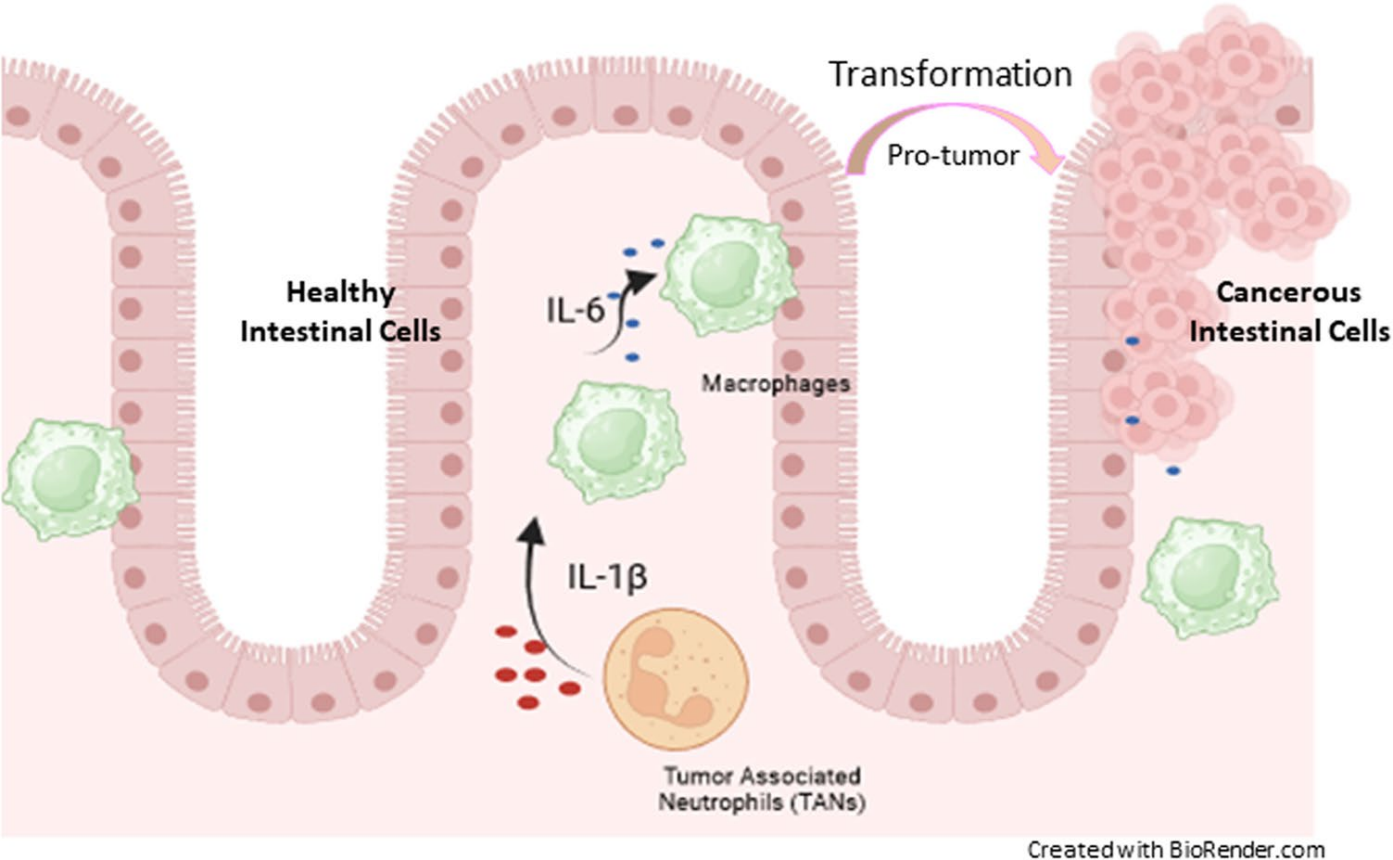
The involvement of macrophages and interleukin (IL-1β) released from Tumor Associated Neutrophil (TANs) for simulating the transformation of colon epithelial cells to cancerous cells

To elaborate on the first hypothesis, the downstream analysis of the pipeline employed criteria such as significant gene expression, promoter methylation, and mRNA expression levels across CRC stages, narrowing 27 hub genes to 8 key biomarkers (CTNNB1, GSK3B, IL-1β, MYC, PXDN, TP53, COL1A1, TGFβ1). Moreover, IL-1β (p-value: 0.038) was linked to poor prognosis, making it both a diagnostic and prognostic marker, as well as a potential immunotherapy target. Second, the literature indicates that non-coding regulators such as miR-548d-3p and miR-559 target ERBB2, with EGFR mutations acting as oncogenic drivers [39, 40]. Moreover, the interplay between PI3K/Akt and Wnt/β-catenin pathways activity often contributes to events related to CRC progression [41, 42]. Some hub genes, such as CTNNB1 and HIF-1α, phosphorylate PI3K/AKT/mTOR signaling networks and upregulate VEGF gene expression by binding to the hypoxia response element, respectively [43, 44]. It is evident that VEGF activates transforming growth factor (TGF)-β pathways and PTEN, together showing an association with PI3K/AKT signaling pathways [45, 46]. Subsequently, high expression of IL-1β, NF-κB, and miR-181a promotes carcinogenesis, while miR-548ac suppresses it by inhibiting TMEM158 [47, 48]. Additionally, STAT5 activates PI3K/Akt and Ras/MAPK pathways via interaction with Gab2 [49]. The coordinated transcriptional and post-transcriptional regulation of hub genes modulates the PI3K/AKT signaling pathway, promoting apoptosis evasion, enhanced cell proliferation, sustained angiogenesis, epithelial-to-mesenchymal transition (EMT) progression, and increased migratory potential. The complete signaling and channelling pathways describing this can be visualized in Fig. 14. Therefore, the proposed hypothesis suggests a cyclical regulatory mechanism where hub genes (GSK3β, AKT1, PI3K, EGFR/ERBB2, PTEN, TP53, TGFβ1), transcription factors (NFκB, STAT5A), and microRNAs (miR-548d-3p, miR-548ac, miR-181a) mutually activate each other, driving the PI3K/AKT pathway. Third, previous research suggests the release of interleukins (IL-1β) in the immune system potentially facilitates the growth and invasion of tumors by stimulating the self-renewal of Cancer Stem Cells (CSC) and EMT [50]. An investigation done by Hai Ping P et al., 2016, linked NF-κB-mediated regulation of IL-1β and miR-181a to PTEN expression, indicating that miR-181a repression promotes colon cancer [47]. Similarly, IL-1β enhances colon cancer cell proliferation by inhibiting GSK3β, thereby activating the Wnt pathway and culminating in tumorigenesis [51]. Additionally, deconvolution analysis revealed a positive correlation between IL-1β expression and infiltration of tumor-associated neutrophils (TANs) and macrophages, contributing to chronic intestinal inflammation in CRC. The early hypothesis proposes a relationship between inflammation and cancer development, a connection substantiated by multiple observed cancer outcomes. Therefore, our findings indicate that interleukins (IL-1β, IL-6, IL-10, TNF) and cytokines (CCL2, CXCL1) drive inflammatory cell infiltration, promoting macrophage activation and IL-6 release, which transforms colon epithelial cells into malignant ones (Fig. 15). The correlation of IL-1β gene with that of other regulatory elements and its association with immune infiltration along with TGF-β, COL1A1, and TP53 genes, have not been documented together in CRC till date. Conclusively, the gene IL-1β and its associated molecules can be proposed as promising diagnostic and prognostic biomarkers and therapeutic targets related to immune infiltration in CRC.

However, this study is subject to several limitations. First, the use of public datasets may not fully capture CRC heterogeneity across populations, and variations in dataset quality could affect reliability. Second, the expression and regulatory mechanism of the derived hub genes require experimental validation to assess their therapeutic potential. Additionally, our focus on gene expression and methylation profiles may overlook the complexities of the tumor microenvironment, including interactions with stromal cells and the extracellular matrix. Next, the exclusion of other omics data types, such as proteomics and metabolomics, limits our understanding of post-transcriptional and metabolic alterations in CRC. Future experiments should include advanced techniques such as multi-omics integration, and multivariant techniques to provide insights into both individual biological entities and pathways critical in disease initiation. Moving forward, further validation through large-scale clinical trials can ensure the robustness of our findings. Finally, the retrospective nature of our study, which utilizes pre-existing datasets, presents certain limitations such as this design may not adequately account for critical confounding factors such as patient comorbidities and varying treatment regimens, potentially impacting the results.

## Conclusion

The study aims to develop promising novel biomarkers from an innovative perspective for enhancing the diagnosis, prognosis, and potential molecular target therapy or immunotherapy for CRC. We reveal eight genes (CTNNB1, GSK3B, IL-1β, MYC, PXDN, TP53, COL1A1, and TGBF1) as diagnostic biomarkers for CRC using an integrative machine learning and statistical approach. Out of these biomarkers, *IL-1β* also exhibited prognostic significance, being associated with survival, stage-specific expression, and disease-free status, and was aberrantly expressed in early-stage CRC in our validation cohort. Along with IL-1β, the genes TGFB1 and COL1A1 also show potential as immunotherapeutic targets due to their positive correlation with immune cell infiltration. The additional involvement of the prognostic analysis and other bioinformatics analysis suggested that TGFB1, COL1A1, and TP53 are biomarkers for early diagnosis and progression of CRC. We hypothesize that elevated IL-1β cytokine level is associated with inflammatory infiltration and is crucial for immunotherapy targeting CRC's advancement. Additionally, the PI3K/Akt signaling pathway is majorly targeted by a few of our hub genes (TGFβ1, AKT1, GSK3β, PTEN, TP53) along with their interplay with the microRNAs (miR 548d-3p, miR 548 ac, and miR 181a) and TFs (NFκB and STAT5A). These candidate molecules and their specific mechanisms are of significant interest to researchers due to their critical roles in diagnostic and prognostic pathways impacting CRC progression.

## Supplementary Information

Additional file 1.

Additional file 2.

## Data Availability

All microarray, RNA-seq and DNA methylation datasets were publically available on GEO database (https://portal.gdc.cancer.gov/) and TCGA-GDC portal (https://www.ncbi.nlm.nih.gov/geo/). All supplementary data are included in this paper. Any information needed to reanalyse the reported findings, can be obtained from the corresponding author upon request.
