# Supplementary material for: IL-1β and associated molecules as prognostic biomarkers linked with immune cell infiltration in colorectal cancer: an integrated statistical and machine learning approach
Source: Discov Oncol. 2025 Feb 28;16:252. doi: 10.1007/s12672-025-01989-3 (PMC11871282; doi:10.1007/s12672-025-01989-3)
Supplement: Supplementary file 1 — Additional file 1. [file 12672_2025_1989_MOESM1_ESM.docx]

**
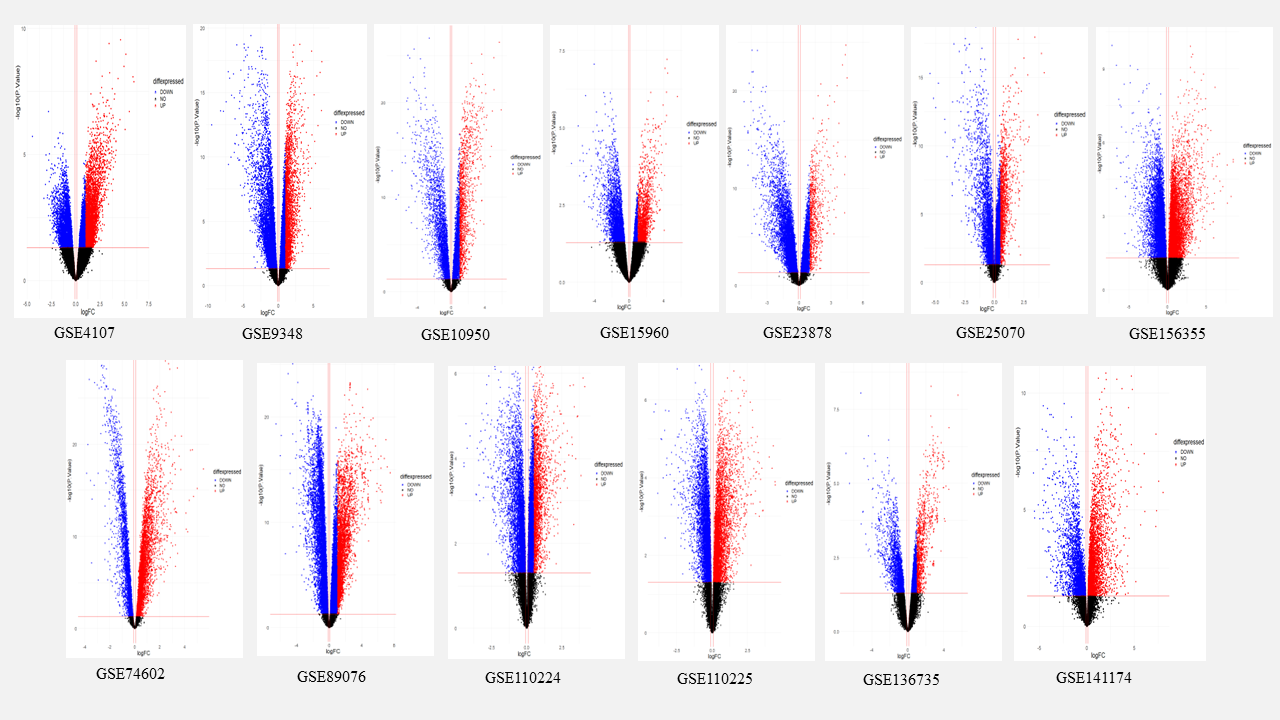
**

**Supplementary Figure 1:** Volcano plot for showing differentially expressed genes in gene expression profiling (microarray) found in the colorectal cancer affected groups to that of the normal group of patients.


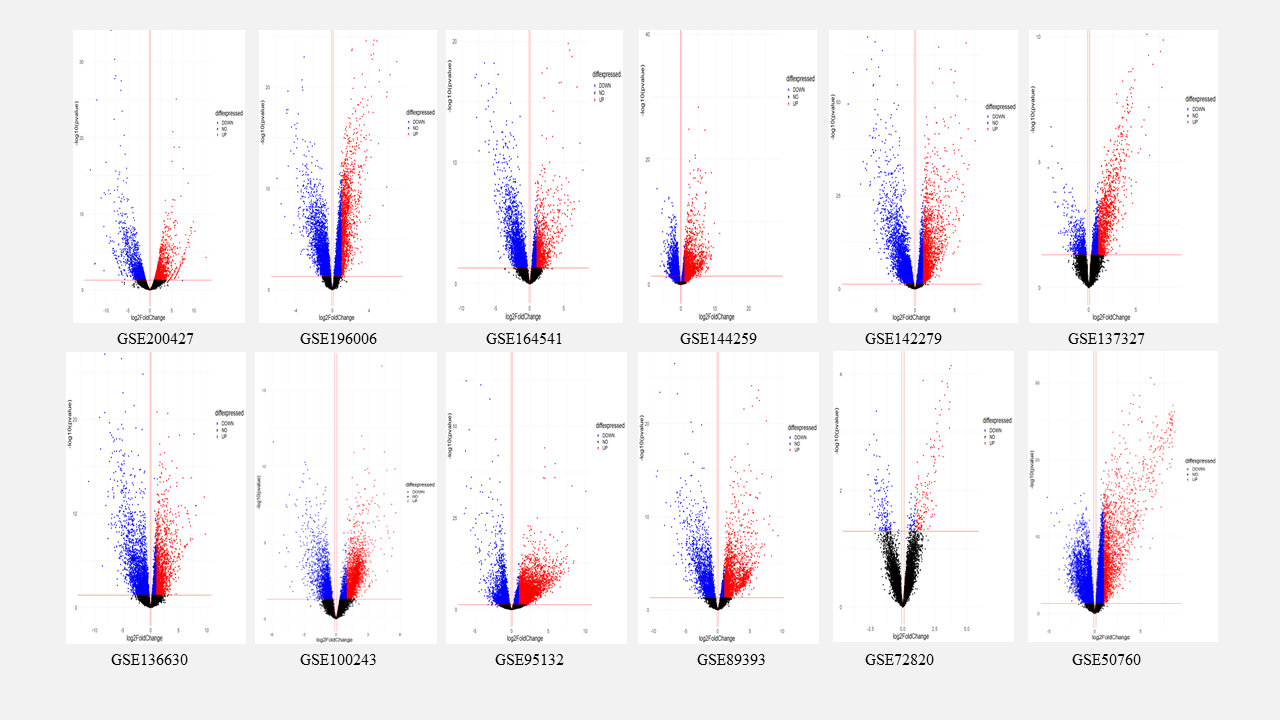


**Supplementary Figure 2:** Volcano plot for showing differentially expressed genes in gene expression profiling (transcriptomics) found in the colorectal cancer affected groups to that of the normal group of patients.


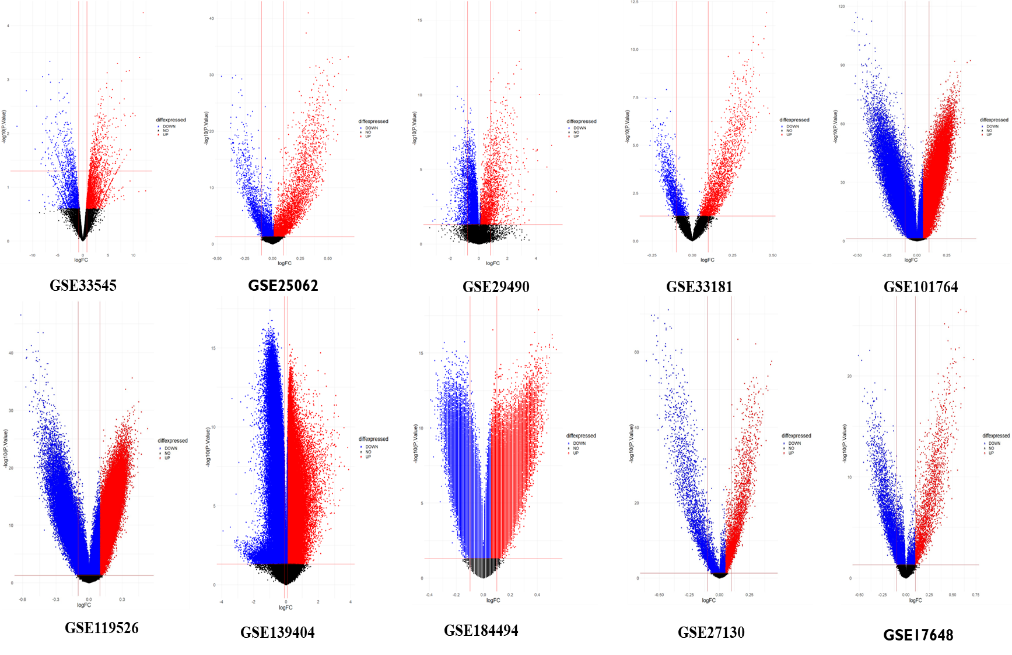

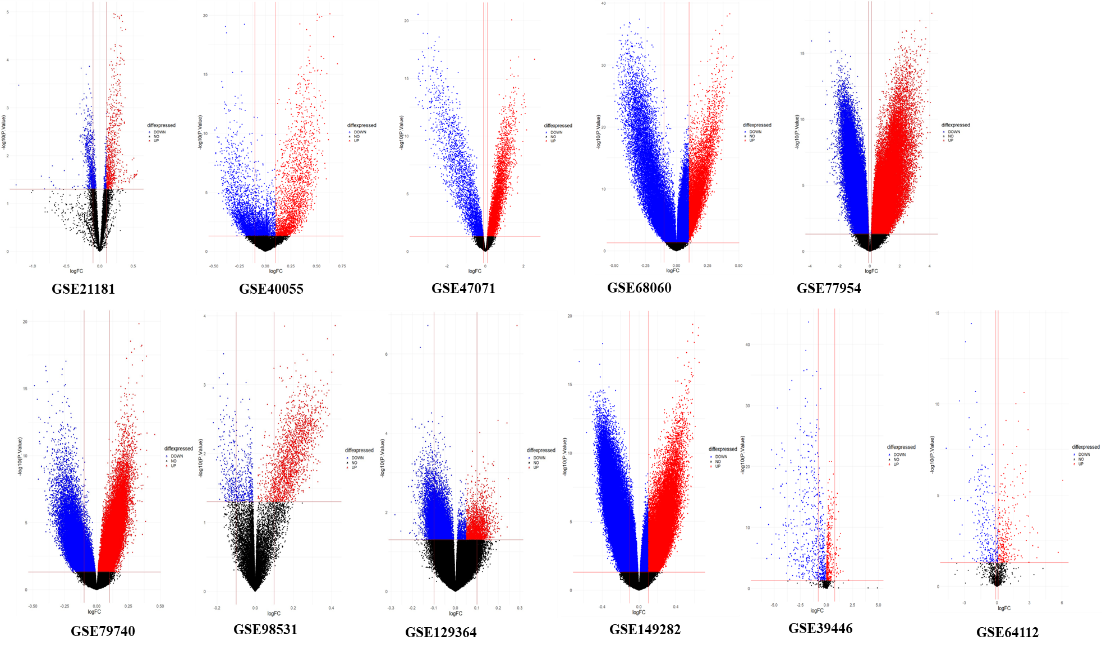


**Supplementary Figure 3**. Volcano plot showing the differentially methylated sites (DMSs) found in the colorectal cancer affected groups to that of the normal group of patients.

**4B**

**4A**


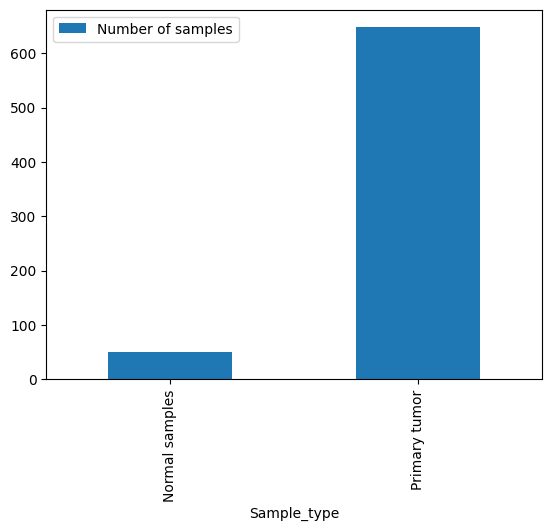

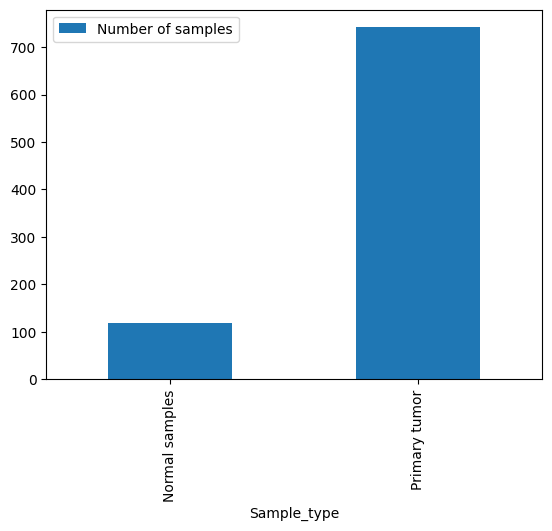


A

**Supplementary Figure 4A:** Distribution of CRC samples for transcriptomics profiling from TCGA database. **Figure 4B:** Distribution of CRC samples for methylation array from TCGA database.


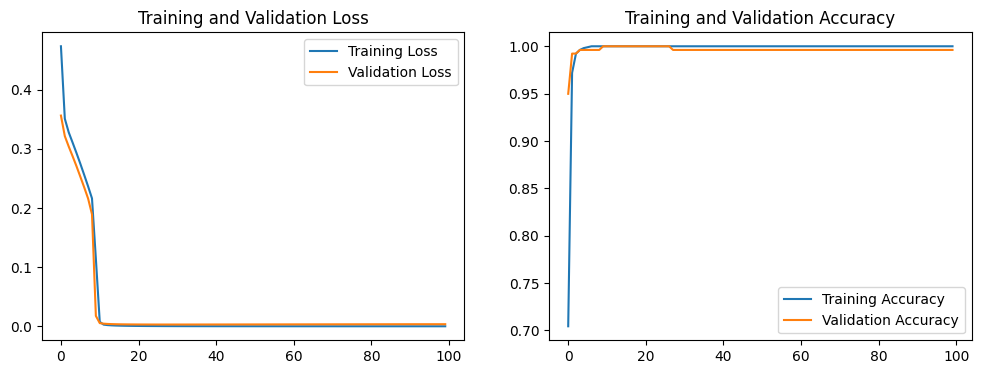


A


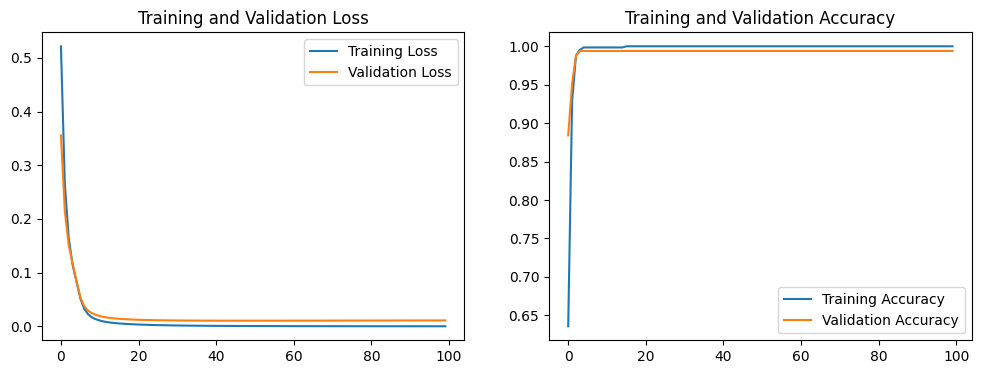

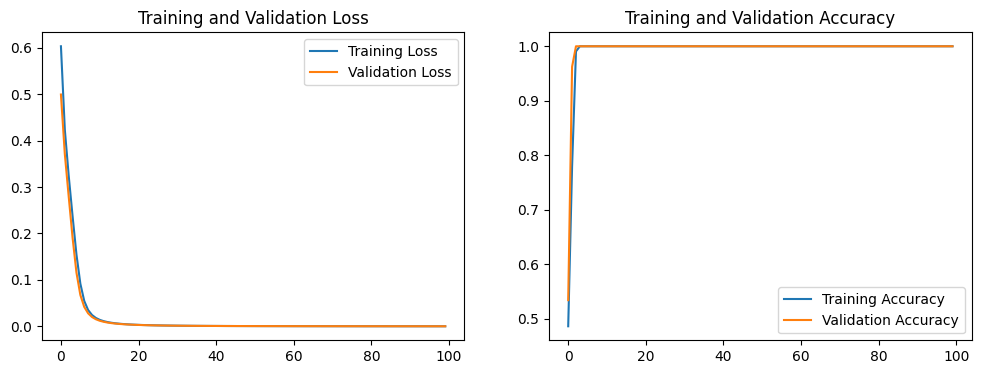


C

B

**Supplementary Figure 5:** The learning plots for the ANN classifier developed for (A) transcriptomics CRC data (B) DNA methylation CRC data (450k), and (C) DNA methylation CRC data (27k).


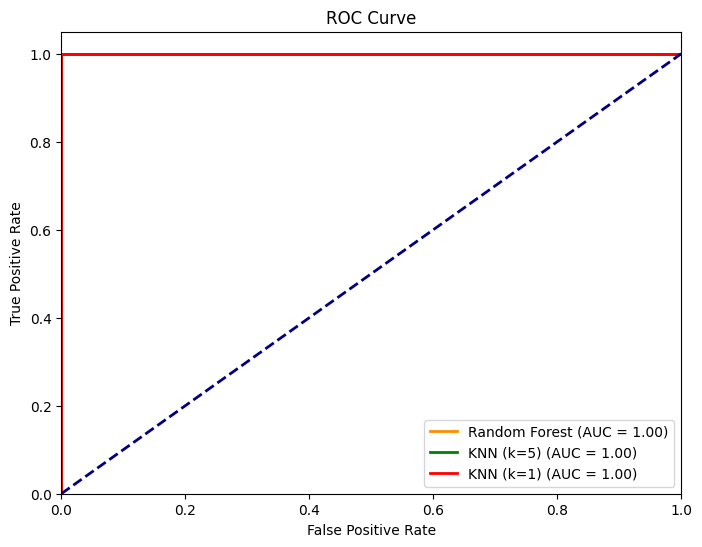


**Supplementary Figure 6:** ROC (Receiver Operating Characteristic) curve graph obtained for evaluating the performance of all developed models including Random Forest and K-Nearest Neighbors (KNN) with *k value* = 1 and *k value* = 0.

| S.no. | GEO Accession no. | Number of samples (case / control) | Country | Year of submission | Reference |
| --- | --- | --- | --- | --- | --- |
| DNA Methylation datasets | | | | | |
| 1. | GSE33545 | 6 samples (3 diseased /3 normal) | China | 2011 | [1] |
| 2. | GSE25062 | 154 samples (125 diseased /29 normal) | USA | 2011 | [2] |
| 3. | GSE29490 | 48 samples (24 diseased /24 normal) | Chicago (USA) | 2012 | [3] |
| 4. | GSE33181 | 48 samples (24 diseased /24 normal) | Chicago (USA) | 2014 | [4] |
| 5. | GSE101764 | 261 Samples (112 diseased /149 normal) | USA | 2017 | [5] |
| 6. | GSE119526 | 96 samples (48 diseased /48 normal) | China | 2023 | [6] |
| 7. | GSE139404 | 60 samples (normal, high grade and low grade) | China | 2019 | [7] |
| 8. | GSE184494 | 48 samples (24 diseased /24 normal) | Germany | 2021 | [8] |
| 9. | GSE17648 | 44 samples (22 diseased /22 normal) | South Korea | 2009 | [9] |
| 10. | GSE21181 | 26 samples (18 diseased /8 normal) | Netherlands | 2011 | [10] |
| 11 | GSE27130 | 248 samples (130 diseased /118 normal) | South Korea | 2011 | [11] |
| 12 | GSE40055 | 42 samples (36 diseased /6 normal) | Denmark | 2013 | [12] |
| 13 | GSE47071 | 89 samples (51 diseased /38 normal) | Cambridge, UK | 2015 | [13] |
| 14 | GSE68060 | 118 samples (82 diseased /36 normal) | Spain | 2015 | [14] |
| 15 | GSE77954 | 48 samples (34 diseased /14 normal) | California, USA | 2016 | [15] |
| 16 | GSE79740 | 54 samples (44 diseased /10 normal) | UK | 2017 | [16] |
| 17 | GSE98531 | 16 samples (8 diseased /8 normal) | Switzerland | 2019 | [17] |
| 18 | GSE129364 | 72 samples (69 diseased /3 normal) | USA | 2019 | [18] |
| 19 | GSE149282 | 24 samples (12 diseased /12 normal) | Malaysia | 2020 | [19] |
| 20 | GSE39446 | 118 samples (91 diseased /28 normal) | Singapore | 2012 | [20] |
| 21 | GSE64112 | 58 samples | Spain | 2015 | [21] |
| Microarray datasets | | | | | |
| 1 | GSE4107 | 22 samples (12 diseased /10 normal) | Singapore | 2007 | [22] |
| 2 | GSE9348 | 82 samples (70 diseased /12 normal) | Singapore | 2010 | [23] |
| 3 | GSE10950 | 48 samples (24 diseased /24 normal) | Japan | 2019 | [24] |
| 4 | GSE15960 | 18 samples (12 diseased /6 normal) | Hungary | 2010 | [25] |
| 5 | GSE23878 | 59 samples (35 diseased /24 normal) | Saudi Arabia | 2010 | [26] |
| 6 | GSE25070 | 52 samples (26 diseased /26 normal) | USA | 2011 | [2] |
| 7 | GSE74602 | 30 samples (15 diseased /15 normal) | Singapore | 2016 | [2] |
| 8 | GSE89076 | 80 samples (41 diseased /39 normal) | Japan | 2017 | [27] |
| 9 | GSE110224 | 34 samples (17 diseased /17 normal) | Greece | 2018 | [28] |
| 10 | GSE110225 | 60 samples (30 diseased /30 normal) | Greece | 2018 | [28] |
| 11 | GSE136735 | 12 samples (6 diseased /6 normal) | China | 2020 | [29] |
| 12 | GSE141174 | 16 samples (8 diseased /8 normal) | Sweden | 2020 | [30] |
| 13 | GSE156355 | 12 samples (6 diseased /6 normal) | China | 2020 | [31] |
| Transcriptomics datasets | | | | | |
| 1 | GSE200427 | 4 samples (2 diseased /2 normal) | China | 2022 | [32] |
| 2 | GSE196006 | 42 samples (21 diseased /21 normal) | USA | 2022 | [33] |
| 3 | GSE164541 | 15 samples (10 diseased /5 normal) | China | 2021 | [34] |
| 4 | GSE144259 | 9 samples (6 diseased /3 normal) | China | 2020 | [35] |
| 5 | GSE142279 | 40 samples (20 diseased /20 normal) | China | 2022 | [36] |
| 6 | GSE137327 | 18 samples (9 diseased /9 normal) | USA | 2019 | [37] |
| 7 | GSE136630 | 12 samples (7 diseased /5 normal) | USA | 2021 | [38] |
| 8 | GSE100243 | 23 samples (16 diseased /7 normal) | Poland | 2019 | [39] |
| 9 | GSE95132 | 31 samples (16 diseased /15 normal) | USA | 2018 | [40] |
| 10 | GSE89393 | 17 samples (11 diseased /6 normal) | Poland | 2018 | [41] |
| 11 | GSE72820 | 14 samples (7 diseased /7 normal) | USA | 2016 | [39] |
| 12 | GSE50760 | 54 samples (36 diseased /18 normal) | South Korea | 2014 | [42] |

**Supplementary Table 1**. Overview of DNA methylation, microarray and Transcriptomics datasets used for the Multiomics data analysis with their respective normal and diseased samples counts.

| S.no | Gene Symbol | Gene name | Description | Reference |
| --- | --- | --- | --- | --- |
| 1. | AKT1 | AKT Serine/Threonine Kinase 1 | AKT/PI3K is a crucial component in various signaling pathways, controlling biological processes like angiogenesis, metabolism, cell survival, and proliferation in both normal and malignant cells. | [43,44] |
| 2. | MYC | MYC Proto-Oncogene | This gene is a proto-oncogene and encodes a nuclear phosphoprotein that plays a role in cell cycle progression, apoptosis and cellular transformation. | [43,44] |
| 3. | CTNNB1 | Catenin Beta 1 | The protein encoded by this gene is part of a complex of proteins that constitute adherens junctions (AJs). Essential for the regulating cell growth and adhesion between cells. | [45,46][47] |
| 4. | STAT3 | Signal Transducer and Activator Of Transcription 3 | This protein mediates the expression of a variety of genes in response to cell stimuli, and thus plays a key role in many cellular processes such as cell growth and apoptosis. | [48,49][50,51] |
| 5. | IL6 | Interleukin 6 | The interaction of IL-6 and its receptor-activated JAKs with following induction/activation of STAT3 through tyrosine phosphorylation and subsequent transcription of target genes is vital in cancer formation. | [52]. |
| 6. | SRC | SRC Proto-Oncogene | This proto-oncogene may play a role in the regulation of embryonic development and cell growth. | [45,46][47] |
| 7. | TP53 | Tumor Protein P53 | This proto-oncogene may play a role in the regulation of embryonic development and cell growth. | [53] |
| 8. | CSF2 | Colony Stimulating Factor 2 | Overexpression of CSF2 in CRC patients may be caused by DNA demethylation. | [54,55][56,57]. |
| 9. | TNF | Tumor Necrosis Factor | This protein is involved in proliferation, differentiation, apoptosis, lipid metabolism, and coagulation. | [58]. |
| 10. | EGFR | Epidermal Growth Factor Receptor | This protein is involved in proliferation, differentiation, apoptosis, lipid metabolism, and coagulation. | [48,49][50,51] |
| 11. | APP | Amyloid Beta Precursor Protein | This gene encodes a cell surface receptor and transmembrane precursor protein that is cleaved by secretases to form a number of peptides. | [54,55][56,57]. |
| 12. | GSK3B | Glycogen Synthase Kinase 3 Beta | GSK-3 Beta is a key kinase involved in cancer cell growth, particularly through the Wnt/β-catenin pathway, and its inhibition could potentially treat pancreatic, colon, and rectal cancers, | [59] |
| 13. | ACTB | Actin Beta | Actions are highly conserved proteins that are involved in cell motility, structure, integrity, and intercellular signaling. | [60,61] |
| 14. | HIF1A | Hypoxia Inducible Factor 1 Subunit Alpha | HIF-1 functions as a master regulator of cellular and systemic homeostatic response to hypoxia by activating transcription of many genes, including those involved in energy metabolism, angiogenesis, apoptosis. | [62,63] |
| 15. | TGFB1 | Transforming Growth Factor Beta 1 | TGF-β attains its tumour suppressive role by regulating cell proliferation, apoptosis and immune cell modulation. | [48,49][50,51] |
| 16. | IL10 | Interleukin 10 | The amount of IL10 in the peritoneal fluid is linked to the extent of tumor spread, and the presence of IL10 in ascitic fluid serves as an indicator for predicting recurrence after surgery aimed at curing stage T4 CRC. It enhances B cell survival, proliferation, and antibody production. | [64] |
| 17. | IL1B | IL1B interleukin 1 beta | IL1B promotes the nuclear translocation of S100A4 protein in gastric cancer cells and the cell's stem-like properties through PI3K pathway. | [62,63] |
| 18. | IFNG | interferon gamma | IFN-γ is a cytokine that protects against diseases by acting directly on target cells or through activation of the host immune system. | [65]. |
| 19. | H3-3B | H3.3 Histone B | Replication-independent histone belonging to the histone H3 family is the protein encoded by H3-3B gene. It has been identified as a key prognostic biomarker for patients with colorectal cancer as it is significantly overexpressed in tumoral tissues. | [66] |
| 20. | PTEN | Phosphatase And Tensin Homolog | It acts as a tumor suppressor by reducing the intracellular concentration of phosphatidylinositol-3,4,5-trisphosphate and inhibiting the PI3K-AKT signaling pathway. | [67] |
| 21. | ALB | Albumin | Reduced serum albumin levels were substantially linked to higher mortality rates from lung, colorectal, prostate, and breast cancer. This is a carrier protein, which act as prognostic factor for several cancers. | [68] |
| 22. | CD8A | CD8 Subunit Alpha | The CD8 antigen, a protein on the surface of many cytotoxic T lymphocytes, facilitates crucial interactions within the immune system, and its presence in inflammatory cells is linked to improved survival outcomes in colorectal cancer patients. | [69] |
| 23. | H6PD | Hexose-6-Phosphate Dehydrogenase/Glucose 1-Dehydrogenase | This gene is upregulated and its high expression is associated with the poor prognosis of colorectal cancer. The enzyme encoded by the H6PD gene is found to be overexpressed in various cancers, and is evidenced to play important role in the proliferation of cancer cells. | [70] |
| 24. | RPS27A | Ribosomal Protein S27a | RPS27a gene exhibits elevated expression levels in conditions such as breast fibroadenomas, as well as colorectal and renal malignancies. It plays a role in enhancing cell growth, managing cell cycle, and suppressing the programmed cell death of leukemia cells. | [71] |
| 25. | COL1A1 | Collagen Type I Alpha 1 Chain | The gene COL1A1 is known to facilitate metastasis of colorectal cancer through its influence on the WNT/PCP signaling pathway. Furthermore, elevated expression is associated with poor prognosis of CRC patients. | [72] |
| 26. | NOTCH1 | Notch Receptor 1 | Notch-1 functions as an oncogene, where its overexpression is associated with CRC diagnosis. The knock down Notch-1 or Notch signaling pathway inhibitor can promote cell apoptosis and suppress the proliferation of cancer cells. | [73] |
| 27. | PXDN | Peroxidasin | PXDN methylation levels as an independent prognostic factor for colorectal tumors. | [74] |

**Supplementary Table 2:** The hub genes with their roles, regulatory mechanisms, and expression patterns in colorectal cancer (CRC).

| S.no. | MicroRNA | Function | Ref | TFs | Function | Ref. |
| --- | --- | --- | --- | --- | --- | --- |
| 1. | **hsa-miR-548ac** | miR-548ac acts as a significant suppressor of laryngeal cancer by promoting cell death through the inhibition of TMEM158, and is referred as potential therapeutic target.  It is downregulated in laryngeal squamous cell carcinoma and pancreatic cancer, suggests its potential role in inhibiting cellular proliferation, inducing apoptotic processes, and metastasis | [75] | **BPTF** | BPTF is regulated by c-Myc that enhances colorectal cancer (CRC) advancement by focusing on Cdc25A. High levels of BPTF expression in CRC were linked to increased cell growth and spread, which could be influenced by using specific siRNAs, shRNAs, or inhibitors targeting BPTF | [76] |
| 2. | **hsa-miR-5692a** | MiR-5692a acts as a cancer-promoting gene in liver cancer progression by modulating HOXD8 expression levels. | [77] | **SRF** | SRSF3 enhances the process of angiogenesis formation in colorectal cancer by modulating SRF, supporting its potential as a therapeutic target. Moreover, one study  suggest that the SRF/miR-214/PTK6/JAK2/STAT3 axis may have utility as a diagnostic and therapeutic target for colon cancer. | [78] |
| 3. | **hsa-miR-548d-3p** | The microRNA miR-548d-3p downregulates RSK4, leading to reduced cell death and increased growth (tumour development), invasion and migration of gastric cancer cells. Furthermore, higher miR-548d-3p levels upregulate the mRNA of CDK2, cyclin A1, cyclin D1, Bax, Bcl-2, N-cadherin, and Vimentin, while reducing E-cadherin mRNA, all through its interaction with RSK4. | [79,80]. | **STAT5A** | STAT5A/B (Signal transducer and activator of transcription 5) is crucial for prostate cancer cell viability and growth, affecting its progression to metastatic disease and also targeting the prolactin receptor-Jak2-Stat5a/b signaling pathway is being used as prostate cancer therapy | [81,82] |
| 4. | **hsa-miR-548bb-3p** | While hsa-miR-548bb-3p and hsa-miR-548h-3p’s direct roles in disease are not documented, their family targets genes like PTEN and LIFR, crucial for ovarian follicle growth | [77] | **ZBTB7A** | ZBTB7A is linked to worse survival in colorectal cancer, suggesting its potential as a prognostic marker and a target for therapy. ZBTB7A(Zinc Finger And BTB Domain Containing 7A), a tumor suppressor in gastric cancer cells, promotes apoptosis, cell migration, and cell cycle arrest, improving patient survival and acting as a transcriptional repressor of glycolytic genes | [83] |
| 5. | **hsa-miR-548z** | hsa-miR-548z is linked to CIP2A overexpression in human cancers, affecting cancer progression and survival rates. Also, the Wnt/β-catenin pathway is enriched within the CIP2A regulatory gene network | [84] | **NFKB1** | The report suggests that activin promotes cancer growth in colorectal cancer primarily through NFkB, which is a key player in cell proliferation in the context of SMAD4-independent signaling.  Also, this transcriptional factor suggests its central role in various biological processes like cell growth and differentiation; tumour development; inflammation, immunity, and apoptosis and also shows high association with dysregulated expression of genes and miRNA, related to colorectal cancer progression | [85,86] |
| 6. | **hsa-miR-548h-3p** | Studies show miR-548-3p acts as a tumor suppressor in lung cancer, suggesting a similar role for hsa-miR-548h-3p, with hsa-miR-548bb-3p also implicated in breast cancer via the PTEN/PI3K/AKT pathway | [87] |  |  |  |

**Supplementary Table 3:** The regulatory molecules (miRNA and TFs) with their functional and regulatory mechanisms in different human diseases.

**References:**

[1] H. Li, Y. Du, D. Zhang, L.N. Wang, C. Yang, B. Liu, W.J. Wang, L. Shi, W.G. Hong, L. Zhang, Y.X. Yang, Identification of novel DNA methylation markers in colorectal cancer using MIRA-based microarrays, Oncol Rep 28 (2012) 99–104. https://doi.org/10.3892/or.2012.1779.

[2] T. Hinoue, D.J. Weisenberger, C.P.E. Lange, H. Shen, H.M. Byun, D. Van Den Berg, S. Malik, F. Pan, H. Noushmehr, C.M. Van Dijk, R.A.E.M. Tollenaar, P.W. Laird, Genome-scale analysis of aberrant DNA methylation in colorectal cancer, Genome Res 22 (2012) 271–282. https://doi.org/10.1101/gr.117523.110.

[3] M.G. Kibriya, M. Raza, F. Jasmine, S. Roy, R. Paul-Brutus, R. Rahaman, C. Dodsworth, M. Rakibuz-Zaman, M. Kamal, H. Ahsan, A genome-wide DNA methylation study in colorectal carcinoma, BMC Med Genomics 4 (2011). https://doi.org/10.1186/1755-8794-4-50.

[4] F. Jasmine, R. Rahaman, S. Roy, M. Raza, R. Paul, M. Rakibuz-Zaman, R. Paul-Brutus, C. Dodsworth, M. Kamal, H. Ahsan, M.G. Kibriya, Interpretation of genome-wide infinium methylation data from ligated DNA in formalin-fixed, paraffin-embedded paired tumor and normal tissue, BMC Res Notes 5 (2012). https://doi.org/10.1186/1756-0500-5-117.

[5] T.M. Barrow, H. Klett, R. Toth, J. Böhm, B. Gigic, N. Habermann, D. Scherer, P. Schrotz-King, S. Skender, C. Abbenhardt-Martin, L. Zielske, M. Schneider, A. Ulrich, P. Schirmacher, E. Herpel, H. Brenner, H. Busch, M. Boerries, C.M. Ulrich, K.B. Michels, Smoking is associated with hypermethylation of the APC 1A promoter in colorectal cancer: the ColoCare Study, Journal of Pathology 243 (2017) 366–375. https://doi.org/10.1002/path.4955.

[6] H. Yu, X. Wang, L. Bai, G. Tang, K.T. Carter, J. Cui, P. Huang, L. Liang, Y. Ding, M. Cai, M. Huang, H. Liu, G. Cao, S. Gallinger, R.K. Pai, D.D. Buchanan, A.K. Win, P.A. Newcomb, J. Wang, W.M. Grady, Y. Luo, DNA methylation profile in CpG-depleted regions uncovers a high-risk subtype of early-stage colorectal cancer, J Natl Cancer Inst 115 (2023) 52–61. https://doi.org/10.1093/jnci/djac183.

[7] J. Fan, J. Li, S. Guo, C. Tao, H. Zhang, W. Wang, Y. Zhang, D. Zhang, S. Ding, C. Zeng, Genome-wide DNA methylation profiles of low- And high-grade adenoma reveals potential biomarkers for early detection of colorectal carcinoma, Clin Epigenetics 12 (2020). https://doi.org/10.1186/s13148-020-00851-3.

[8] N. Patil, M.L. Abba, C. Zhou, S. Chang, T. Gaiser, J.H. Leupold, H. Allgayer, Changes in methylation across structural and microrna genes relevant for progression and metastasis in colorectal cancer, Cancers (Basel) 13 (2021). https://doi.org/10.3390/cancers13235951.

[9] J. Liu, H. Li, L. Sun, Z. Wang, C. Xing, Y. Yuan, Aberrantly methylated-differentially expressed genes and pathways in colorectal cancer, Cancer Cell Int 17 (2017). https://doi.org/10.1186/s12935-017-0444-4.

[10] E.H.J. Van Roon, N.F.C.C. De Miranda, M.P. Van Nieuwenhuizen, E.J. De Meijer, M. Van Puijenbroek, P.S. Yan, T.H.M. Huang, T. Van Wezel, H. Morreau, J.M. Boer, Tumour-specific methylation of PTPRG intron 1 locus in sporadic and Lynch syndrome colorectal cancer, European Journal of Human Genetics 19 (2011) 307–312. https://doi.org/10.1038/ejhg.2010.187.

[11] Y.H. Kim, H.C. Lee, S.Y. Kim, Y. Il Yeom, K.J. Ryu, B.H. Min, D.H. Kim, H.J. Son, P.L. Rhee, J.J. Kim, J.C. Rhee, H.C. Kim, H.K. Chun, W.M. Grady, Y.S. Kim, Epigenomic analysis of aberrantly methylated genes in colorectal cancer identifies genes commonly affected by epigenetic alterations, Ann Surg Oncol 18 (2011) 2338–2347. https://doi.org/10.1245/s10434-011-1573-y.

[12] B. Øster, L. Linnet, L.L. Christensen, K. Thorsen, H. Ongen, E.T. Dermitzakis, J. Sandoval, S. Moran, M. Esteller, T.F. Hansen, P. Lamy, S. Laurberg, T.F. Ørntoft, C.L. Andersen, Non-CpG island promoter hypomethylation and miR-149 regulate the expression of SRPX2 in colorectal cancer, Int J Cancer 132 (2013) 2303–2315. https://doi.org/10.1002/ijc.27921.

[13] S. Uribe-Lewis, R. Stark, T. Carroll, M.J. Dunning, M. Bachman, Y. Ito, L. Stojic, S. Halim, S.L. Vowler, A.G. Lynch, B. Delatte, E.J. de Bony, L. Colin, M. Defrance, F. Krueger, A.L. Silva, R. ten Hoopen, A.E.K. Ibrahim, F. Fuks, A. Murrell, 5-hydroxymethylcytosine marks promoters in colon that resist DNA hypermethylation in cancer, Genome Biol 16 (2015). https://doi.org/10.1186/s13059-015-0605-5.

[14] J. Wei, G. Li, S. Dang, Y. Zhou, K. Zeng, M. Liu, Discovery and validation of hypermethylated markers for colorectal cancer, Dis Markers 2016 (2016). https://doi.org/10.1155/2016/2192853.

[15] X. Qu, T. Sandmann, H. Frierson, L. Fu, E. Fuentes, K. Walter, K. Okrah, C. Rumpel, C. Moskaluk, S. Lu, Y. Wang, R. Bourgon, E. Penuel, A. Pirzkall, L. Amler, M.R. Lackner, J. Tabernero, G.M. Hampton, O. Kabbarah, Integrated genomic analysis of colorectal cancer progression reveals activation of EGFR through demethylation of the EREG promoter, Oncogene 35 (2016) 6403–6415. https://doi.org/10.1038/onc.2016.170.

[16] M.A. Alvi, M.B. Loughrey, P. Dunne, S. McQuaid, R. Turkington, M.A. Fuchs, C. McGready, V. Bingham, B. Pang, W. Moore, P. Maxwell, M. Lawler, J.A. James, G.I. Murray, R.H. Wilson, M. Salto-Tellez, Molecular profiling of signet ring cell colorectal cancer provides a strong rationale for genomic targeted and immune checkpoint inhibitor therapies, Br J Cancer 117 (2017) 203–209. https://doi.org/10.1038/bjc.2017.168.

[17] F. Noreen, T. Küng, L. Tornillo, H. Parker, M. Silva, S. Weis, G. Marra, R. Rad, K. Truninger, P. Schär, DNA methylation instability by BRAF-mediated TET silencing and lifestyle-exposure divides colon cancer pathways, Clin Epigenetics 11 (2019). https://doi.org/10.1186/s13148-019-0791-1.

[18] D. Fiedler, D. Hirsch, N. El Hajj, H.H. Yang, Y. Hu, C. Sticht, I. Nanda, S. Belle, J. Rueschoff, M.P. Lee, T. Ried, T. Haaf, T. Gaiser, Genome-wide DNA methylation analysis of colorectal adenomas with and without recurrence reveals an association between cytosine-phosphate-guanine methylation and histological subtypes, Genes Chromosomes Cancer 58 (2019) 783–797. https://doi.org/10.1002/gcc.22787.

[19] M. Ishak, R. Baharudin, I.M. Rose, I. Sagap, L. Mazlan, Z.A.M. Azman, N. Abu, R. Jamal, L.H. Lee, N.S. Ab Mutalib, Genome-wide open chromatin methylome profiles in colorectal cancer, Biomolecules 10 (2020). https://doi.org/10.3390/biom10050719.

[20] P.W. Ang, M. Loh, N. Liem, P.L. Lim, F. Grieu, A. Vaithilingam, C. Platell, W.P. Yong, B. Iacopetta, R. Soong, Comprehensive profiling of DNA methylation in colorectal cancer reveals subgroups with distinct clinicopathological and molecular features, 2010. http://www.biomedcentral.com/1471-2407/10/227.

[21] K. Torabi, R. Miró, N. Fernández-Jiménez, I. Quintanilla, L. Ramos, E. Prat, J. del Rey, N. Pujol, J. Keith Killian, P.S. Meltzer, P.L. Fernández, T. Ried, J.J. Lozano, J. Camps, I. Ponsa, Patterns of somatic uniparental disomy identify novel tumor suppressor genes in colorectal cancer, Carcinogenesis 36 (2015) 1103–1110. https://doi.org/10.1093/carcin/bgv115.

[22] Y. Hong, S.H. Kok, W.E. Kong, Y.C. Peh, A susceptibility gene set for early onset colorectal cancer that integrates diverse signaling pathways: Implication for tumorigenesis, Clinical Cancer Research 13 (2007) 1107–1114. https://doi.org/10.1158/1078-0432.CCR-06-1633.

[23] Y. Hong, T. Downey, K.W. Eu, P.K. Koh, P.Y. Cheah, A “metastasis-prone” signature for early-stage mismatch-repair proficient sporadic colorectal cancer patients and its implications for possible therapeutics, Clin Exp Metastasis 27 (2010) 83–90. https://doi.org/10.1007/s10585-010-9305-4.

[24] X. Jiang, J. Tan, J. Li, S. Kivimäe, X. Yang, L. Zhuang, P.L. Lee, M.T.W. Chan, L.W. Stanton, E.T. Liu, B.N.R. Cheyette, Q. Yu, DACT3 Is an Epigenetic Regulator of Wnt/β-Catenin Signaling in Colorectal Cancer and Is a Therapeutic Target of Histone Modifications, Cancer Cell 13 (2008) 529–541. https://doi.org/10.1016/j.ccr.2008.04.019.

[25] O. Galamb, S. Spisák, F. Sipos, K. Tóth, N. Solymosi, B. Wichmann, T. Krenács, G. Valcz, Z. Tulassay, B. Molnár, Reversal of gene expression changes in the colorectal normal-adenoma pathway by NS398 selective COX2 inhibitor, Br J Cancer 102 (2010) 765–773. https://doi.org/10.1038/sj.bjc.6605515.

[26] S. Uddin, M. Ahmed, A. Hussain, J. Abubaker, N. Al-Sanea, A. AbdulJabbar, L.H. Ashari, S. Alhomoud, F. Al-Dayel, Z. Jehan, P. Bavi, A.K. Siraj, K.S. Al-Kuraya, Genome-wide expression analysis of Middle Eastern colorectal cancer reveals FOXM1 as a novel target for cancer therapy, American Journal of Pathology 178 (2011) 537–547. https://doi.org/10.1016/j.ajpath.2010.10.020.

[27] K. Satoh, S. Yachida, M. Sugimoto, M. Oshima, T. Nakagawa, S. Akamoto, S. Tabata, K. Saitoh, K. Kato, S. Sato, K. Igarashi, Y. Aizawa, R. Kajino-Sakamoto, Y. Kojima, T. Fujishita, A. Enomoto, A. Hirayama, T. Ishikawa, M.M. Taketo, Y. Kushida, R. Haba, K. Okano, M. Tomita, Y. Suzuki, S. Fukuda, M. Aoki, T. Soga, Global metabolic reprogramming of colorectal cancer occurs at adenoma stage and is induced by MYC, Proc Natl Acad Sci U S A 114 (2017) E7697–E7706. https://doi.org/10.1073/pnas.1710366114.

[28] E.I. Vlachavas, E. Pilalis, O. Papadodima, D. Koczan, S. Willis, S. Klippel, C. Cheng, L. Pan, C. Sachpekidis, A. Pintzas, V. Gregoriou, A. Dimitrakopoulou-Strauss, A. Chatziioannou, Radiogenomic Analysis of F-18-Fluorodeoxyglucose Positron Emission Tomography and Gene Expression Data Elucidates the Epidemiological Complexity of Colorectal Cancer Landscape, Comput Struct Biotechnol J 17 (2019) 177–185. https://doi.org/10.1016/j.csbj.2019.01.007.

[29] R. Shen, P. Li, B. Li, B. Zhang, L. Feng, S. Cheng, Identification of Distinct Immune Subtypes in Colorectal Cancer Based on the Stromal Compartment, Front Oncol 9 (2020). https://doi.org/10.3389/fonc.2019.01497.

[30] L. Olsson, M.L. Hammarström, A. Israelsson, G. Lindmark, S. Hammarström, Allocating colorectal cancer patients to different risk categories by using a five-biomarker mRNA combination in lymph node analysis, PLoS One 15 (2020). https://doi.org/10.1371/journal.pone.0229007.

[31] H.W. Sun, J. Chen, W.C. Wu, Y.Y. Yang, Y.T. Xu, X.J. Yu, H.T. Chen, Z. Wang, X.J. Wu, L. Zheng, Retinoic acid synthesis deficiency fosters the generation of polymorphonuclear myeloid-derived suppressor cells in colorectal cancer, Cancer Immunol Res 9 (2021) 20–33. https://doi.org/10.1158/2326-6066.CIR-20-0389.

[32] Y. Jiang, X. Wang, L. Li, J. He, Q. Jin, D. Long, C. Liu, W. Zhou, K. Liu, A systematic analysis of C5ORF46 in gastrointestinal tumors as a potential prognostic and immunological biomarker, Front Genet 13 (2022). https://doi.org/10.3389/fgene.2022.926943.

[33] O.M. Marx, M.M. Mankarious, M.A. Eshelman, W. Ding, W.A. Koltun, G.S. Yochum, Transcriptome Analyses Identify Deregulated MYC in Early Onset Colorectal Cancer, Biomolecules 12 (2022). https://doi.org/10.3390/biom12091223.

[34] Q. Hong, B. Li, X. Cai, Z. Lv, S. Cai, Y. Zhong, B. Wen, Transcriptomic Analyses of the Adenoma-Carcinoma Sequence Identify Hallmarks Associated With the Onset of Colorectal Cancer, Front Oncol 11 (2021). https://doi.org/10.3389/fonc.2021.704531.

[35] Q. Ji, L. Zhou, H. Sui, L. Yang, X. Wu, Q. Song, R. Jia, R. Li, J. Sun, Z. Wang, N. Liu, Y. Feng, X. Sun, G. Cai, Y. Feng, J. Cai, Y. Cao, G. Cai, Y. Wang, Q. Li, Primary tumors release ITGBL1-rich extracellular vesicles to promote distal metastatic tumor growth through fibroblast-niche formation, Nat Commun 11 (2020). https://doi.org/10.1038/s41467-020-14869-x.

[36] J. Zhang, Q. Gao, S. Hou, X. Chi, M. Zheng, Q. Zhang, H. Shan, X. Zhang, C. Kang, Role of PAX6, TRPA1, BCL11B, MCOLN2, CUX1, EMX1 in colorectal cancer and osteosarcoma, Medicine (United States) 103 (2024) E37056. https://doi.org/10.1097/MD.0000000000037056.

[37] S. Nomiri, R. Hoshyar, E. Chamani, Z. Rezaei, F. Salmani, P. Larki, T. Tavakoli, F. gholipour, N.J. Tabrizi, A. Derakhshani, M. Santarpia, T. Franchina, O. Brunetti, N. Silvestris, H. Safarpour, Prediction and validation of GUCA2B as the hub-gene in colorectal cancer based on co-expression network analysis: In-silico and in-vivo study, Biomedicine and Pharmacotherapy 147 (2022). https://doi.org/10.1016/j.biopha.2022.112691.

[38] E. Orouji, A.T. Raman, A.K. Singh, A. Sorokin, E. Arslan, A.K. Ghosh, J. Schulz, C.J. Terranova, S. Jiang, M. Tang, M. Maitituoheti, P. Barrodia, Y. Jiang, S.C. Callahan, K.J. Tomczak, Z. Jiang, J.S. Davis, S. Ghosh, H.M. Lee, L. Reyes-Uribe, K. Chang, Y. Liu, H. Chen, A. Azhdarnia, J.S. Morris, E. Vilar, K.S. Carmon, S. Kopetz, K. Rai, Chromatin State Dynamics Confers Specific Therapeutic Strategies in Enhancer Subtypes of Colorectal Cancer HHS Public Access, Gut 71 (2022) 938–949. https://doi.org/10.5281/zenodo.819971.

[39] K. Urh, N. Zidar, E. Boštjančič, Bioinformatics Analysis of RNA-seq Data Reveals Genes Related to Cancer Stem Cells in Colorectal Cancerogenesis, Int J Mol Sci 23 (2022). https://doi.org/10.3390/ijms232113252.

[40] M.P. Hanley, M.A. Hahn, A.X. Li, X. Wu, J. Lin, J. Wang, A.H. Choi, Z. Ouyang, Y. Fong, G.P. Pfeifer, T.J. Devers, D.W. Rosenberg, Genome-wide DNA methylation profiling reveals cancer-associated changes within early colonic neoplasia, Oncogene 36 (2017) 5035–5044. https://doi.org/10.1038/onc.2017.130.

[41] K. Goryca, M. Kulecka, A. Paziewska, M. Dabrowska, M. Grzelak, M. Skrzypczak, K. Ginalski, A. Mroz, A. Rutkowski, K. Paczkowska, M. Mikula, J. Ostrowski, Exome scale map of genetic alterations promoting metastasis in colorectal cancer, BMC Genet 19 (2018). https://doi.org/10.1186/s12863-018-0673-0.

[42] S.K. Kim, S.Y. Kim, J.H. Kim, S.A. Roh, D.H. Cho, Y.S. Kim, J.C. Kim, A nineteen gene-based risk score classifier predicts prognosis of colorectal cancer patients, Mol Oncol 8 (2014) 1653–1666. https://doi.org/10.1016/j.molonc.2014.06.016.

[43] S. Rennoll, Regulation of MYC gene expression by aberrant Wnt/β-catenin signaling in colorectal cancer , World J Biol Chem 6 (2015) 290. https://doi.org/10.4331/wjbc.v6.i4.290.

[44] S. Dihlmann, M. Kloor, C. Fallsehr, M. von Knebel Doeberitz, Regulation of AKT1 expression by beta-catenin/Tcf/Lef signaling in colorectal cancer cells, Carcinogenesis 26 (2005) 1503–1512. https://doi.org/10.1093/carcin/bgi120.

[45] C. Gao, Y. Wang, R. Broaddus, L. Sun, F. Xue, W. Zhang, Exon 3 mutations of CTNNB1 drive tumorigenesis: a review, n.d. www.impactjournals.com/oncotarget.

[46] S. Razak, N. Bibi, J.A. Dar, T. Afsar, A. Almajwal, Z. Parveen, S. Jahan, Screening and computational analysis of colorectal associated non-synonymous polymorphism in CTNNB1 gene in Pakistani population, BMC Med Genet 20 (2019). https://doi.org/10.1186/s12881-019-0911-y.

[47] W. Jin, Regulation of Src family kinases during colorectal cancer development and its clinical implications, Cancers (Basel) 12 (2020). https://doi.org/10.3390/cancers12051339.

[48] A.M. Krasinskas, EGFR Signaling in Colorectal Carcinoma, Patholog Res Int 2011 (2011) 1–6. https://doi.org/10.4061/2011/932932.

[49] A.J. Pellatt, L.E. Mullany, J.S. Herrick, L.C. Sakoda, R.K. Wolff, W.S. Samowitz, M.L. Slattery, The TGFβ-signaling pathway and colorectal cancer: Associations between dysregulated genes and miRNAs, J Transl Med 16 (2018). https://doi.org/10.1186/s12967-018-1566-8.

[50] A.N. Gargalionis, K.A. Papavassiliou, A.G. Papavassiliou, Targeting STAT3 signaling pathway in colorectal cancer, Biomedicines 9 (2021). https://doi.org/10.3390/biomedicines9081016.

[51] H. Xiong, J. Hong, W. Du, Y.W. Lin, L.L. Ren, Y.C. Wang, W.Y. Su, J.L. Wang, Y. Cui, Z.H. Wang, J.Y. Fang, Roles of STAT3 and ZEB1 proteins in E-cadherin down-regulation and human colorectal cancer epithelial-mesenchymal transition, Journal of Biological Chemistry 287 (2012) 5819–5832. https://doi.org/10.1074/jbc.M111.295964.

[52] M.J. Waldner, S. Foersch, M.F. Neurath, Interleukin-6 - A key regulator of colorectal cancer development, Int J Biol Sci 8 (2012) 1248–1253. https://doi.org/10.7150/ijbs.4614.

[53] M. Michel, L. Kaps, A. Maderer, P.R. Galle, M. Moehler, The role of p53 dysfunction in colorectal cancer and its implication for therapy, Cancers (Basel) 13 (2021). https://doi.org/10.3390/cancers13102296.

[54] R. Ji, C. Wu, J. Yao, J. Xu, J. Lin, H. Gu, M. Fu, X. Zhang, Y. Li, X. Zhang, IGF2BP2-meidated m6A modification of CSF2 reprograms MSC to promote gastric cancer progression, Cell Death Dis 14 (2023). https://doi.org/10.1038/s41419-023-06163-7.

[55] M. Sielska, P. Przanowski, M. Pasierbińska, K. Wojnicki, K. Poleszak, B. Wojtas, D. Grzeganek, A. Ellert-Miklaszewska, M.C. Ku, H. Kettenmann, B. Kaminska, Tumour-derived CSF2/granulocyte macrophage colony stimulating factor controls myeloid cell accumulation and progression of gliomas, Br J Cancer 123 (2020) 438–448. https://doi.org/10.1038/s41416-020-0862-2.

[56] H.N. Lee, M.S. Jeong, S.B. Jang, Molecular characteristics of amyloid precursor protein (App) and its effects in cancer, Int J Mol Sci 22 (2021). https://doi.org/10.3390/ijms22094999.

[57] F.R.P. Dewi, T. Domoto, M. Hazawa, A. Kobayashi, T. Douwaki, T. Minamoto, R.W. Wong, Colorectal cancer cells require glycogen synthase kinase-3β for sustaining mitosis via translocated promoter region (TPR)-dynein interaction, 2018. www.impactjournals.com/oncotarget.

[58] O.A. Al Obeed, K.A. Alkhayal, A. Al Sheikh, A.M. Zubaidi, M.A. Vaali-Mohammed, R. Boushey, J.H. Mckerrow, M.H. Abdulla, Increased expression of tumor necrosis factor-α is associated with advanced colorectal cancer stages, World J Gastroenterol 20 (2014) 18390–18396. https://doi.org/10.3748/wjg.v20.i48.18390.

[59] R.J. Vidri, T.L. Fitzgerald, GSK-3: An important kinase in colon and pancreatic cancers, Biochim Biophys Acta Mol Cell Res 1867 (2020). https://doi.org/10.1016/j.bbamcr.2019.118626.

[60] L. Xu, H. Luo, R. Wang, W.W. Wu, J.N. Phue, R.F. Shen, H. Juhl, L. Wu, W.L. Alterovitz, V. Simonyan, L. Pelosof, A.S. Rosenberg, Novel reference genes in colorectal cancer identify a distinct subset of high stage tumors and their associated histologically normal colonic tissues, BMC Med Genet 20 (2019). https://doi.org/10.1186/s12881-019-0867-y.

[61] Y. Gu, S. Tang, Z. Wang, L. Cai, H. Lian, Y. Shen, Y. Zhou, A pan-cancer analysis of the prognostic and immunological role of β-actin (ACTB) in human cancers, Bioengineered 12 (2021) 6166–6185. https://doi.org/10.1080/21655979.2021.1973220.

[62] Y. Baba, K. Nosho, K. Shima, N. Irahara, A.T. Chan, J.A. Meyerhardt, D.C. Chung, E.L. Giovannucci, C.S. Fuchs, S. Ogino, HIF1A overexpression is associated with poor prognosis in a cohort of 731 colorectal cancers, American Journal of Pathology 176 (2010) 2292–2301. https://doi.org/10.2353/ajpath.2010.090972.

[63] J. Paredes, J. Zabaleta, J. Garai, P. Ji, S. Imtiaz, M. Spagnardi, J. Alvarado, L. Li, M. Akadri, K. Barrera, M. Munoz-Sagastibelza, R. Gupta, M. Alshal, M. Agaronov, H. Talus, X. Wang, J.M. Carethers, J.L. Williams, L.A. Martello, Immune-Related Gene Expression and Cytokine Secretion Is Reduced Among African American Colon Cancer Patients, Front Oncol 10 (2020). https://doi.org/10.3389/fonc.2020.01498.

[64] S.Y. Jeong, B.G. Jeon, J.E. Kim, R. Shin, H.S. Ahn, H. Jin, S.C. Heo, Interleukin 10 level in the peritoneal cavity is a prognostic marker for peritoneal recurrence of T4 colorectal cancer, Sci Rep 11 (2021). https://doi.org/10.1038/s41598-021-88653-2.

[65] W. Du, T.L. Frankel, M. Green, W. Zou, IFNγ signaling integrity in colorectal cancer immunity and immunotherapy, Cell Mol Immunol 19 (2022) 23–32. https://doi.org/10.1038/s41423-021-00735-3.

[66] H.A. Ayoubi, F. Mahjoubi, R. Mirzaei, Investigation of the human H3.3B (H3F3B) gene expression as a novel marker in patients with colorectal cancer, J Gastrointest Oncol 8 (2017) 64–69. https://doi.org/10.21037/jgo.2016.12.12.

[67] M.M. Georgescu, Pten tumor suppressor network in PI3K-Akt pathway control, Genes Cancer 1 (2010) 1170–1177. https://doi.org/10.1177/1947601911407325.

[68] J. Zhu, L. Li, Y. Duan, Y. Wu, X. Wang, Prognostic role of pre-treatment serum ALB in Patients with oropharyngeal cancer: A retrospective cohort study, Front Oncol 12 (2022). https://doi.org/10.3389/fonc.2022.924210.

[69] J. Kasurinen, J. Hagström, T. Kaprio, I. Beilmann-Lehtonen, C. Haglund, C. Böckelman, Tumor-associated CD3- and CD8-positive immune cells in colorectal cancer: The additional prognostic value of CD8+-to-CD3+ ratio remains debatable, Tumor Biology 44 (2022) 37–52. https://doi.org/10.3233/TUB-211571.

[70] M. Tsachaki, N. Mladenovic, H. Štambergová, J. Birk, A. Odermatt, Hexose-6-phosphate dehydrogenase controls cancer cell proliferation and migration through pleiotropic effects on the unfolded-protein response, calcium homeostasis, and redox balance, FASEB Journal 32 (2018) 2690–2705. https://doi.org/10.1096/fj.201700870RR.

[71] H. Wang, J. Yu, L. Zhang, Y. Xiong, S. Chen, H. Xing, Z. Tian, K. Tang, H. Wei, Q. Rao, M. Wang, J. Wang, RPS27a promotes proliferation, regulates cell cycle progression and inhibits apoptosis of leukemia cells, Biochem Biophys Res Commun 446 (2014) 1204–1210. https://doi.org/10.1016/j.bbrc.2014.03.086.

[72] Z. Zhang, Y. Wang, J. Zhang, J. Zhong, R. Yang, COL1A1 promotes metastasis in colorectal cancer by regulating the WNT/PCP pathway, Mol Med Rep 17 (2018) 5037–5042. https://doi.org/10.3892/mmr.2018.8533.

[73] R. Jackstadt, S.R. van Hooff, J.D. Leach, X. Cortes-Lavaud, J.O. Lohuis, R.A. Ridgway, V.M. Wouters, J. Roper, T.J. Kendall, C.S. Roxburgh, P.G. Horgan, C. Nixon, C. Nourse, M. Gunzer, W. Clark, A. Hedley, O.H. Yilmaz, M. Rashid, P. Bailey, A. V. Biankin, A.D. Campbell, D.J. Adams, S.T. Barry, C.W. Steele, J.P. Medema, O.J. Sansom, Epithelial NOTCH Signaling Rewires the Tumor Microenvironment of Colorectal Cancer to Drive Poor-Prognosis Subtypes and Metastasis, Cancer Cell 36 (2019) 319-336.e7. https://doi.org/10.1016/j.ccell.2019.08.003.

[74] J.Q. Su, P.Y. Lai, P.H. Hu, J.M. Hu, P.K. Chang, C.Y. Chen, J.J. Wu, Y.J. Lin, C.A. Sun, T. Yang, C.H. Hsu, H.C. Lin, Y.C. Chou, Differential DNA methylation analysis of SUMF2, ADAMTS5, and PXDN provides novel insights into colorectal cancer prognosis prediction in Taiwan, World J Gastroenterol 28 (2022) 825–839. https://doi.org/10.3748/wjg.v28.i8.825.

[75] F. Song, Y. Yang, J. Liu, MicroRNA-548ac induces apoptosis in laryngeal squamous cell carcinoma cells by targeting transmembrane protein 158, Oncol Lett 20 (2020). https://doi.org/10.3892/ol.2020.11930.

[76] P. Guo, S. Zu, S. Han, W. Yu, G. Xue, X. Lu, H. Lin, X. Zhao, H. Lu, C. Hua, X. Wan, L. Ru, Z. Guo, H. Ge, K. Lv, G. Zhang, W. Deng, C. Luo, W. Guo, BPTF inhibition antagonizes colorectal cancer progression by transcriptionally inactivating Cdc25A, Redox Biol 55 (2022). https://doi.org/10.1016/j.redox.2022.102418.

[77] F. Deng, J. Mu, C. Qu, F. Yang, X. Liu, X. Zeng, X. Peng, A Novel Prognostic Model of Endometrial Carcinoma Based on Clinical Variables and Oncogenomic Gene Signature, Front Mol Biosci 7 (2021). https://doi.org/10.3389/fmolb.2020.587822.

[78] Y. Chen, M. Yang, F. Meng, Y. Zhang, M. Wang, X. Guo, J. Yang, H. Zhang, H. Zhang, J. Sun, W. Wang, SRSF3 Promotes Angiogenesis in Colorectal Cancer by Splicing SRF, Front Oncol 12 (2022). https://doi.org/10.3389/fonc.2022.810610.

[79] H. Liang, C. Hu, X. Lin, Z. He, Z. Lin, J. Dai, Mir-548d-3p promotes gastric cancer by targeting rsk4, Cancer Manag Res 12 (2020) 13325–13337. https://doi.org/10.2147/CMAR.S278691.

[80] miR‐548d‐3p inhibits the invasion and migration of gastric cancer cells by targeting GKN1, (n.d.).

[81] S.H. Tan, A. Dagvadorj, F. Shen, L. Gu, Z. Liao, J. Abdulghani, Y. Zhang, E.P. Gelmann, T. Zellweger, Z. Culig, T. Visakorpi, L. Bubendorf, R.A. Kirken, J. Karras, M.T. Nevalainen, Transcription factor Stat5 synergizes with androgen receptor in prostate cancer cells, Cancer Res 68 (2008) 236–248. https://doi.org/10.1158/0008-5472.CAN-07-2972.

[82] W. Du, Y.C. Wang, J. Hong, W.Y. Su, Y.W. Lin, R. Lu, H. Xiong, J.Y. Fang, STAT5 isoforms regulate colorectal cancer cell apoptosis via reduction of mitochondrial membrane potential and generation of reactive oxygen species, J Cell Physiol 227 (2012) 2421–2429. https://doi.org/10.1002/jcp.22977.

[83] L. Wang, M.X. Zhang, M.F. Zhang, Z.W. Tu, ZBTB7A functioned as an oncogene in colorectal cancer, BMC Gastroenterol 20 (2020). https://doi.org/10.1186/s12876-020-01456-z.

[84] M.M. Tarek, A. Yahia, M.M. El-Nakib, M. Elhefnawi, Integrative assessment of CIP2A overexpression and mutational effects in human malignancies identifies possible deleterious variants, Comput Biol Med 139 (2021). https://doi.org/10.1016/j.compbiomed.2021.104986.

[85] A. Jana, N.L. Krett, G. Guzman, A. Khalid, O. Ozden, J.J. Staudacher, J. Bauer, S.H. Baik, T. Carroll, C. Yazici, B. Jung, NFkB is essential for activin-induced colorectal cancer migration via upregulation of PI3K-MDM2 pathway, 2017. www.impactjournals.com/oncotarget.

[86] The NF-κB signalling pathway in colorectal cancer_associations between dysregulated gene and miRNA expression , (n.d.).

[87] M. Yadollahi-Farsani, Z. Amini-Farsani, F. Moayedi, N. Khazaei, H. Yaghoobi, MiR-548k suppresses apoptosis in breast cancer cells by affecting PTEN/PI3K/AKT signaling pathway, IUBMB Life 75 (2023) 97–116. https://doi.org/10.1002/iub.2688.
